# RUNX1 prevents oestrogen-mediated AXIN1 suppression and β-catenin activation in ER-positive breast cancer

**DOI:** 10.1038/ncomms10751

**Published:** 2016-02-26

**Authors:** Nyam-Osor Chimge, Gillian H. Little, Sanjeev K. Baniwal, Helty Adisetiyo, Ying Xie, Tian Zhang, Andie O'Laughlin, Zhi Y. Liu, Peaches Ulrich, Anthony Martin, Paulette Mhawech-Fauceglia, Matthew J. Ellis, Debu Tripathy, Susan Groshen, Chengyu Liang, Zhe Li, Dustin E. Schones, Baruch Frenkel

**Affiliations:** 1 Department of Medicine, Keck School of Medicine of the University of Southern California, Los Angeles, California 90033, USA; 2 Institute for Genetic Medicine, Keck School of Medicine of the University of Southern California, Los Angeles, California 90033, USA; 3 Division of Genetics, Department of Medicine, Brigham and Women's Hospital, Harvard Medical School, Boston, Massachusetts 02115, USA; 4 Department of Molecular Microbiology and Immunology, Keck School of Medicine of the University of Southern California, Los Angeles, California 90033, USA; 5 Department of Pathology, Keck School of Medicine of the University of Southern California, Los Angeles, California 90033, USA; 6 Smith Breast Center, Baylor College of Medicine, Houston, Texas 77030, USA; 7 Department of Breast Medical Oncology, The University of Texas MD Anderson Cancer Center, Houston, Texas 77030, USA; 8 Department of Preventive Medicine, Keck School of Medicine of the University of Southern California, Los Angeles, California 90033, USA; 9 USC/Norris Comprehensive Cancer Center, Keck School of Medicine of the University of Southern California, Los Angeles, California 90033, USA; 10 Department of Diabetes Complications and Metabolism, Beckman Research Institute, City of Hope, Duarte, California 91010, USA; 11 Department of Orthopedic Surgery, Keck School of Medicine of the University of Southern California, Los Angeles, California 90033, USA; 12 Department of Biochemistry and Molecular Biology, Keck School of Medicine of the University of Southern California, Los Angeles, California 90033, USA

## Abstract

Recent high-throughput studies revealed recurrent *RUNX1* mutations in breast cancer, specifically in oestrogen receptor-positive (ER^+^) tumours. However, mechanisms underlying the implied RUNX1-mediated tumour suppression remain elusive. Here, by depleting mammary epithelial cells of RUNX1 *in vivo* and *in vitro,* we demonstrate combinatorial regulation of *AXIN1* by RUNX1 and oestrogen. RUNX1 and ER occupy adjacent elements in *AXIN1*'s second intron, and RUNX1 antagonizes oestrogen-mediated *AXIN1* suppression. Accordingly, RNA-seq and immunohistochemical analyses demonstrate an ER-dependent correlation between RUNX1 and AXIN1 in tumour biopsies. RUNX1 loss in ER^+^ mammary epithelial cells increases β-catenin, deregulates mitosis and stimulates cell proliferation and expression of stem cell markers. However, it does not stimulate LEF/TCF, *c-Myc* or *CCND1,* and it does not accelerate G1/S cell cycle phase transition. Finally, RUNX1 loss-mediated deregulation of β-catenin and mitosis is ameliorated by AXIN1 stabilization *in vitro*, highlighting AXIN1 as a potential target for the management of ER^+^ breast cancer.

## Abstract (web summary)

The tumour suppressor RUNX1 is often lost or mutated in oestrogen receptor-positive breast cancers. In this study, the authors demonstrate that the loss of RUNX1 unleashes oestrogen-mediated inhibition of AXIN1, a negative regulator of β-catenin, resulting in β-catenin signalling-mediated cancer cell proliferation and mitosis deregulation.

## Graphical abstract

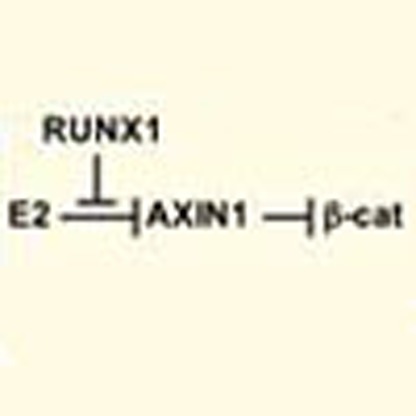

Beside their developmental roles, particularly in haematopoiesis, skeletogenesis and neurogenesis, the three mammalian RUNX transcription factors have been assigned both oncogenic and tumour suppressor functions in a variety of neoplastic diseases^1^,^2^,^3^. In breast cancer, *RUNX3* is frequently inactivated by promoter hypermethylation or protein mislocalization, its expression inversely correlates with disease progression^4^,^5^, and its haploinsufficiency in mice promotes mammary ductal carcinoma^6^. Mechanistically, RUNX3 (as well as RUNX2) antagonize ERα^6^,^7^,^8^,^9^. RUNX2, however, is better known for its pro-metastatic activity in breast and other carcinomas^3^,^10^. Little attention has been paid thus far to the potential roles of RUNX1 in breast cancer. Recent studies, however, demonstrate that it is the predominant *RUNX* family member expressed in mammary epithelial cells^2^, and growing evidence suggests context-dependent dual roles for RUNX1 in breast cancer progression^2^,^11^,^12^,^13^,^14^,^15^,^16^,^17^. In particular, three independent studies of breast cancer patient cohorts have recently reported recurrent somatic mutations and/or deletions of *RUNX1*, as well as *CBFB* that encodes an obligate co-activator of RUNX1 (refs 18, 19, 20). Here, we demonstrate that RUNX1 antagonizes oestrogen-mediated inhibition of *AXIN1* expression, shedding light on its breast cancer suppression role.

Nearly two-thirds of all breast cancer cases belong to the ER^+^ luminal subtype^21^. ERα, which plays important physiological roles in mammary epithelial cell growth and differentiation during puberty and pregnancy, can acquire deleterious functions that promote breast carcinogenesis^22^,^23^,^24^. This is associated with changes to ERα-mediated transcriptional stimulation or repression, attributable, in part, to increased ERα levels or alterations to modifying transcription factors such as FOXA, GATA, AP2γ and their associated co-regulators^25^,^26^,^27^,^28^. The present work calls attention to the ERα-interacting transcription factor RUNX1 (ref. 29). It suggests that loss of RUNX1 in breast cancer facilitates ERα-mediated suppression of *AXIN1*, resulting in aberrant β-catenin signalling.

β-Catenin plays pivotal roles in cancer, primarily attributable to its role in canonical Wnt signalling. Upon Wnt pathway stimulation, a constitutively active β-catenin destruction complex is disassembled, allowing β-catenin to accumulate and ultimately activate LEF/TCF target genes such as *CCND1, c-Myc, AXIN2* and *LEF1* itself^30^,^31^. The β-catenin destruction complex contains, among others, the scaffold proteins AXIN1 and APC (adenomatous polyposis coli), as well as glycogen synthase kinase 3α/β (GSK3α/β), which phosphorylate and mark β-catenin for proteasomal degradation^30^,^32^. In addition, β-catenin resides in the centrosome, where it regulates microtubule dynamics and bipolar mitotic spindle formation^33^,^34^,^35^. At the centrosome, β-catenin is phosphorylated by another kinase, NEK2, but is protected from degradation^36^. Despite its established oncogenic role in general, several issues regarding the role of β-catenin in ER^+^ breast cancer remain to be elucidated. For instance, expression of β-catenin/TCF-regulated genes, both endogenous Wnt targets and reporter constructs, is poorly correlated with Wnt-driven mammary epithelial cell transformation that occur either spontaneously or experimentally^37^,^38^. In particular, increased expression of *c-Myc* and *CCND1*, implicated in Wnt-driven loss of G1/S cell cycle control in colon cancer, is either absent or dispensable in many cases of ER^+^ breast cancer^37^,^38^. Furthermore, it is unclear if and how oestrogen signalling may regulate β-catenin in breast cancer.

In this study, by depleting RUNX1 *in vitro* and *in vivo*, we expose a link between oestrogen and β-catenin in ER^+^ breast cancer, that is, RUNX1-gated oestrogen-mediated *AXIN1* transcriptional repression. Furthermore, we present evidence that deregulation of β-catenin in RUNX1-deficient ER^+^ breast cancer cells is associated with compromised mitotic checkpoint control, accelerated cell proliferation and increased expression of stem cell markers. Our work marks AXIN1 as a potential therapeutic target to remedy deregulation of β-catenin in ER^+^ breast cancer tumours that have lost RUNX1 function through somatic mutations or other mechanisms.

## Results

### RUNX1 loss deregulates β-catenin

**Figure 1:**
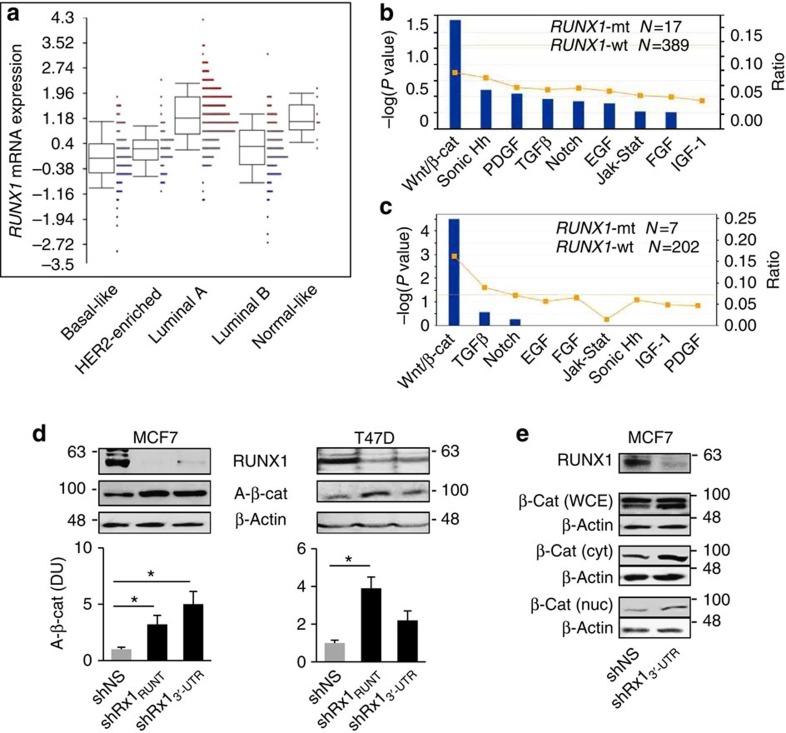
Upregulation of β-catenin in RUNX1-deficient breast cancer. (**a**) RUNX1 mRNA expression in the five major breast cancer subtypes in the breast cancer cohort of TCGA^20^. Expression levels are significantly different between the subtypes (*P*=6.8e−38 by analysis of variance). Boxes represent the 25% to 75% quartiles, lines within boxes represent the median levels and whiskers represent the non-outlier range. (**b**) Genes differentially expressed in ER^+^ tumours with mutant versus wild-type *RUNX1* in the breast cancer patient cohort of TCGA^20^ (Supplementary Data 1) were interrogated using Ingenuity Pathways Analysis (IPA) for annotations related to major developmental signalling pathways. Line graph represents fold enrichment, and statistical significance (bars) was calculated by Fisher's exact test as implemented in the IPA software. (**c**) IPA analysis was performed as in **b** for the differentially expressed genes (Supplementary Data 2) in RUNX1-mutant tumours in the breast cancer patient cohort of Ellis *et al.*^18^ (**d**) Top: representative western blot analyses of the indicated proteins in MCF7 and T47D cells expressing either a nonspecific shRNA (shNS) or shRNAs targeting the Runt domain (shRx1_RUNT_) or the 3′-UTR (shRx1_3′-UTR_) of RUNX1. Bottom: western blots from three independent experiments were scanned using the ImageJ software, and bar graphs represent mean densitometric values (±s.e.m.) for normalized A-β-cat corrected for β-actin. *P<0.05 by *t*-test. (**e**) Western blot analysis of total β-catenin in whole-cell extracts (WCE), as well as cytoplasmic (cyt) and nuclear (nuc) fractions of MCF7 cells expressing the shNS or the shRx1_3′-UTR_ RNAs.

Expression of *RUNX1* in the breast cancer cohort of The Cancer Genome Atlas (TCGA)^20^ varied considerably among individual tumours, with strong dependence on tumour subtype (Fig. 1a). *RUNX1* mutations, mostly in the Runt DNA-binding domain^2^, were identified in 18 of the overall 524 tumours in this cohort, and 17 of them were within the group of 406 ER^+^ tumours^20^. In pursuit of molecular mechanisms contributing to its implied tumour suppressor activity in ER^+^ breast cancer, we performed pathway analysis of genes differentially expressed in the ER^+^ tumours with versus without *RUNX1* mutations in TCGA, as well as in the ER^+^ breast cancer cohort of Ellis *et.al*.^18^ Annotations associated with genes differentially expressed in each of these cohorts (Fig. 1b,c) or in both cohorts (Supplementary Fig. 1) were most significantly related to Wnt/β-catenin signalling. The three gene lists and their inclusion criteria are provided in Supplementary Data 1–3, respectively.

Prompted by the data mining results, we next assessed the effect of RUNX1 silencing on β-catenin in the MCF7 and T47D cell culture models. Both of them represent the ER^+^ luminal A breast cancer subtype, in which *RUNX1* is most highly expressed (Fig. 1a), attributable in part to promoter hypomethylation (Supplementary Fig. 2). RUNX1 silencing with shRNAs that target either its RUNT domain (shRx1_RUNT_) or its 3′-untranslated region (3′-UTR; shRx1_3′-UTR_) upregulated active β-catenin (A-β-cat) levels in both cell lines (Fig. 1d), and increased cytoplasmic and nuclear β-catenin was confirmed by western blot analysis of the respective MCF7 cell fractions (Fig. 1e).

### RUNX1 loss promotes cell growth and stem cell markers

**Figure 2:**
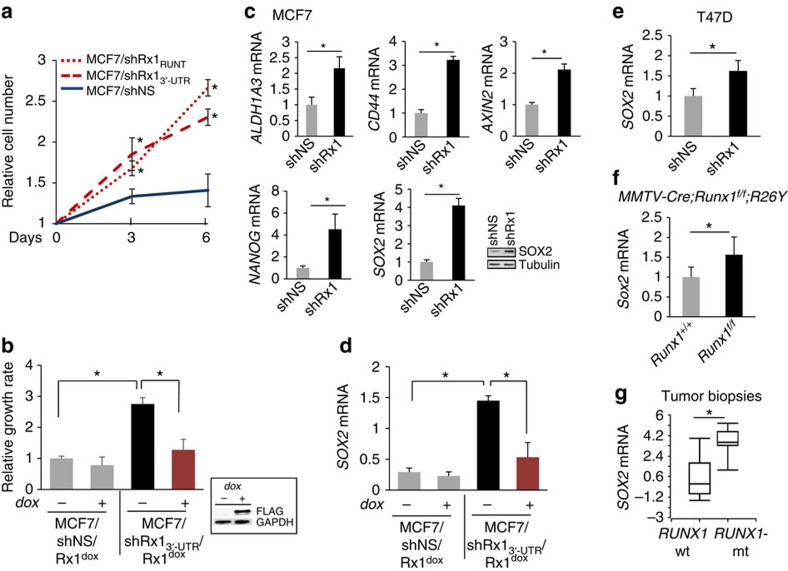
Increased proliferation and expression of stem cell markers in RUNX1-depleted mammary epithelial cells *in vitro* and *in vivo*. (**a**) MCF7 cells expressing nonspecific (NS) or the indicated RUNX1-targeting shRNAs were plated and their growth rate was assessed by MTT assays on days 3 and 6. (**b**) RUNX1 was silenced with shRx1_3′-UTR_ as in Fig. 1d,e, and was re-expressed from a dox-inducible vector as demonstrated by the western blot in the inset. Cell growth was assessed as in **a** and bars represent the increase in MTT values between day 3 and day 6. (**c**) MCF7 cells expressing a nonspecific shRNA (shNS) or shRx1_3′-UTR_ were subjected to RT–qPCR analysis of the indicated stem cell markers. Expression of Sox2 was also assessed by western blot analysis. (**d**) RT–qPCR analysis of *SOX2* in MCF7 cells in which RUNX1 was silenced and then restored as in **b**. (**e**–**g**) Comparisons of *SOX2* mRNA expression between (**e**) RUNX1-depleted versus control T47D cells; (**f**) mammary luminal epithelial cells from *MMTV-Cre;Runx*^*f/f*^*;R26Y* versus control *MMTV-Cre;Runx1*^*+/+*^*;R26Y* mice based on our microarray data in GSE47377 (ref. 13); (**g**) *RUNX1*-mutant (*n*=7) versus *RUNX1*-WT (*n*=202) breast cancer tumours based on our microarray database^18^, where boxes represent the 25th–75th percentile range, horizontal lines within boxes represent the median values and whiskers extend to the minimum and maximum values. Where applicable, data represent mean±s.e.m. of triplicate experiments. *P<0.05 by *t*-test (**a**–**f**) or by Mann–Whitney test (**g**).

Deregulation of β-catenin has been linked to cancer cell proliferation in general and cancer stem cells in particular^39^,^40^,^41^. Accordingly, RUNX1 knockdown with either shRx1_RUNT_ or shRx1_3′-UTR_ resulted in increased MCF7 breast cancer cell proliferation (Fig. 2a). Furthermore, conditional re-expression of RUNX1 in MCF7/shRx1_3′-UTR_ cells using a dox-inducible system normalized their growth rate (Fig. 2b). In addition, RUNX1 silencing was associated with upregulation of the stem cell markers *ALDH1A3, CD44*, *AXIN2, NANOG* and *SOX2* (refs 42, 43, 44, 45, 46; Fig. 2c), and RUNX1 restoration normalized *SOX2* mRNA levels (Fig. 2d). Similarly in T47D breast cancer cells, RUNX1 knockdown increased cell growth rate^13^ and *SOX2* expression (Fig. 2e). Moreover, mRNA profiles of *Runx1*-deficient mammary luminal epithelial cells from *MMTV-Cre;Runx1*^*f/f*^ versus control mice (GSE 47377) indicated increased expression of *Sox2* in response to Runx1 loss (Fig. 2f). Finally, as shown in Fig. 2g, *SOX2* mRNA was markedly elevated in biopsies from *RUNX1*-mutant versus *RUNX1*-WT human primary breast tumours in the clinical cohort of Ellis *et al.*^18^ Thus, deregulation of β-catenin in RUNX1-deficient ER^+^ breast cancer might contribute to disease progression by promoting cell growth in general and expansion of a stem cell-like population in particular.

### RUNX1 regulates *AXIN1*

**Figure 3:**
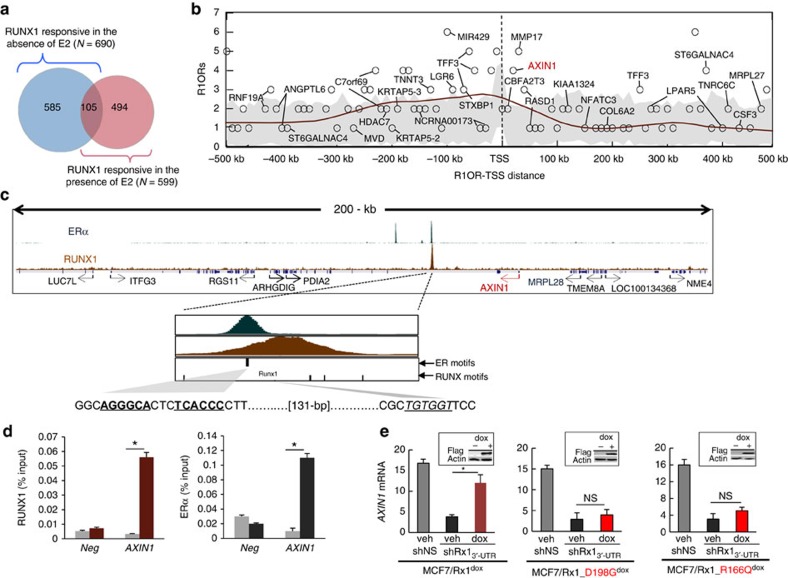
Recruitment of RUNX1 and ERα to adjacent elements in *AXIN1*. (**a**) The genome-wide response to RUNX1 was determined as described in the ‘Methods' section by comparing the mRNA profiles of MCF7/shRx1_3′-UTR_ and MCF7/shNS cells maintained in CSS±E2. The Venn diagram describes the RUNX1 responsiveness (number of genes with fold change >1.5 and *P*<0.05) in the presence and absence of E2. (**b**) RUNX1-occupied regions (R1ORs) were determined by ChIP-seq analysis of MCF7 cells and mapped with respect to the TSSs of the 599 genes that responded to RUNX1 in the presence of E2. For 111 out of these 599 genes, R1ORs were found between positions −500 kb and +500 kb of the respective TSSs, and these R1ORs (a total of 176) were enumerated in 10-kb bins. Grey area represents the results of 1,000 iterations with control random gene sets. The brown line is a density curve of R1OR occurrence generated by LOESS fitting to R1OR counts (*α*=0.25). Gene names are depicted in cases where R1ORs overlapped with ER-occupied regions deduced from GSE14644 (ref. 47). (**c**) Screen shots of RUNX1 and ERα ChIP-seq data for the *AXIN1* locus, with zoom-in on ERα and RUNX1 peaks at a region co-occupied by the two transcription factors. (**d**) ChIP-qPCR confirmation of RUNX1 and ERα occupancy at the second intron of *AXIN1*. Data obtained with antibodies against RUNX1 (left) and ERα (right) are represented by brown and black bars, respectively, and data obtained with control IgG is represented by grey bars. *P<0.05 by *t*-test. (**e**) Endogenous *RUNX1* in MCF7 cells was silenced by shRx1_3′-UTR_ and the indicated wild-type or mutant FLAG-tagged RUNX1 proteins were induced by dox as in Fig. 2b,d. *AXIN1* expression was assessed by RT–qPCR. Data was corrected for 18S RNA and represent mean±s.e.m. from triplicate experiments. *P<0.05 by *t*-test. Insets represent western blot analyses of the exogenous FLAG-tagged proteins.

In pursuit of RUNX1 target genes in ER^+^ breast cancer, which may mediate the regulation of β-catenin levels, we determined both the RUNX1 transcriptome and its cistrome in MCF7 cells by mRNA profiling and ChIP-seq analysis, respectively. We first compared global mRNA expression in cells expressing shRx1_3′-UTR_ versus cells expressing a nonspecific hairpin RNA (shNS) as in Figs 1e and 2. Because recurrent *RUNX1* mutations are specific to ER^+^ tumours^20^, we determined the differentially expressed genes both in the presence and absence of estradiol (E2), and turned our attention to 599 genes that responded to RUNX1 knockdown in the presence of E2 (Fig. 3a, Supplementary Data 4). We then profiled RUNX1 locations with ChIP-seq to identify putative direct RUNX1 targets, those that not only respond to RUNX1 (in the presence of E2) but also physically associate with RUNX1. As shown in Fig. 3b, there was enrichment of RUNX1-occupied regions (R1ORs) near the transcription start sites (TSSs) of the RUNX1-responsive genes, likely related to short-range direct transcriptional regulation. We next interrogated an MCF7 ERα ChIP-seq data set^47^ to identify putative RUNX1-responsive enhancers that also recruited ERα, potentially accounting for dependence of the RUNX1 response on oestrogen. Of the 176 R1ORs present between positions −500 kb and +500 kb relative to the TSSs of the 599 RUNX1-reponsive genes, 36 genes (named in Fig. 3b) were also occupied by ERα. Among the regions co-occupied by RUNX1 and ERα was the second intron of *AXIN1* (Fig. 3b,c), encoding a pivotal regulatory component of the β-catenin destruction complex. Co-occupancy of this region by RUNX1 and ERα was validated by ChIP-quantitative PCR (ChIP-qPCR; Fig. 3d). RT–qPCR (qPCR with reverse transcription) analysis confirmed the downregulation of *AXIN1* expression upon RUNX1 silencing (Fig. 3e). Furthermore, conditional induction (by dox treatment) of wild-type RUNX1, but not RUNX1 mutants with the amino acid substitutions D198G or R166Q found in human breast cancers^18^,^20^, restored *AXIN1* mRNA expression (Fig. 3e).

### RUNX1 prevents oestrogen-mediated inhibition of *AXIN1*

**Figure 4:**
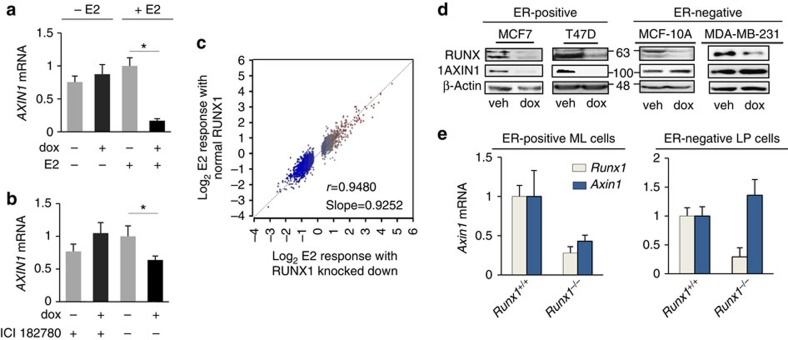
RUNX1 prevents oestrogen-mediated *AXIN1* repression. (**a**) MCF7/shRx1_RUNT_^dox^ cells were maintained in 10% charcoal-stripped serum for 48 h, treated as indicated for the following 48 h, and *AXIN1* mRNA levels were measured by RT–qPCR and corrected for 18S RNA (mean±s.e.m. of three independent experiments). (**b**) MCF7/shRx1_RUNT_^dox^ cells in 10% complete serum were treated as indicated for 48 h, and AXIN1 mRNA levels were measured by RT–qPCR and corrected for 18S RNA (mean±s.e.m. of three independent experiments). *P<0.05 by *t*-test. (**c**) Scatter plot of the global E2 responsiveness in the presence (*y* axis) versus absence (*x* axis) of RUNX1 in MCF7 cells. (**d**) The indicated ER^+^ (left) and ER^−^ (right) mammary epithelial cell lines were engineered with the dox-inducible shRx1_3′-UTR_ lentiviral vector and treated with dox for 4 days before western blot analysis of the indicated proteins. (**e**) RT–qPCR results for *Axin1* and *Runx1* from predominantly ER^+^ mature luminal (ML) mammary epithelial cells (left) and predominantly ER^−^ luminal progenitor (LP) cells (right) isolated from *RUNX1*-knockout and control mammary glands as described in the ‘Methods' section.

We next addressed the dependence of the RUNX1-*AXIN1* axis on oestrogen signalling using cell and animal experimental models, as well as clinical data mining. First, we assessed by RT–qPCR the effect of dox-mediated RUNX1 silencing on *AXIN1* expression in MCF7/shRx1^dox^ cultures maintained in charcoal-stripped serum (CSS) with or without added E2. As shown in Fig. 4a, RUNX1 silencing in the absence of oestrogens did not itself affect *AXIN1* expression. However, RUNX1 silencing in the presence of E2 resulted in the suppression of *AXIN1*. Second, we tested the dependence of the RUNX1-*AXIN1* axis on oestrogens in MCF7/shRx1^dox^ cells cultured in complete (oestrogen-containing) serum (as in Figs 1 and 2) with or without the ER antagonist/downregulator ICI 182780. As shown in Fig. 4b, dox-mediated RUNX1 silencing resulted in decreased *AXIN1* expression in the absence but not in the presence of ICI 182780. These results suggest that *AXIN1* is negatively regulated by oestrogen signalling, and that this negative regulation is denied by RUNX1 (Fig. 4a,b). Notably, the combinatorial regulation of *AXIN1* by RUNX1 and E2 is unusual, because the global transcriptional response to RUNX1 knockdown is generally independent on E2 (Fig. 4c). In addition, unlike the global transcriptional response to RUNX2 overexpression^7^,^9^,^48^, the global transcriptional response to RUNX1 overexpression is generally independent of E2 (Supplementary Fig. 3).

We further examined the dependence of the RUNX1-*AXIN1* axis on oestrogen signalling *in vitro* by comparing the effect of dox-mediated RUNX1 silencing on *AXIN1* expression in ER^+^ versus ER^−^ mammary epithelial cell lines. As demonstrated by western blot analysis, dox-mediated silencing of *RUNX1* with either shRx1_3′-UTR_ (Fig. 4d) or shRx1_RUNT_ (Supplementary Fig. 4) resulted in the downregulation of AXIN1 expression in the ER^+^ MCF7 and T47D cells, but not in the ER^−^ MDA-MB-231 or MCF10A cells.

We next set to test the effect of Runx1 on *Axin1* mRNA expression in ER^+^ versus ER^−^ murine mammary epithelial cells *in vivo*. Our strategy was to isolate mature luminal cells (ML; predominantly ER-positive) and luminal progenitor cells (LP; predominantly ER-negative) from *MMTV-Cre;Runx1*^*f/f*^ and control mice, and compare the two cell populations in terms of the effect of *Runx1* ablation on *Axin1* expression. However, because, as reported previously^13^, the ML cell population was virtually lost in *MMTV-Cre;Runx1*^*f/f*^ mice, we isolated Runx1-depleted and control ML cells from mice with additional ablation of *Rb1*, in which the ML cell population was restored despite the absence of *Runx1*. As shown in Fig. 4e, *Runx1* ablation resulted in decreased *Axin1* expression in the predominantly ER^+^ ML cells but not in the predominantly ER^-^ LP cells, again suggesting E2-dependent regulation of *AXIN1* by RUNX1. Thus, multiple experimental approaches *in vitro* and *in vivo* indicate combinatorial regulation of *AXIN1* by RUNX1 and oestrogens in both normal and transformed mammary epithelial cells. These results implicate *AXIN1* suppression in E2-driven breast carcinogenesis, containment of which accounts for the tumour suppressor activity of RUNX1 in ER^+^ breast cancer. Dependence of the RUNX1-*AXIN1* axis on oestrogens may explain the observation of recurrent RUNX1 somatic mutations in ER^+^ but not ER^−^ breast cancer tumours^20^.

### Association between RUNX1 and AXIN1 in ER^+^ breast cancer

**Figure 5:**
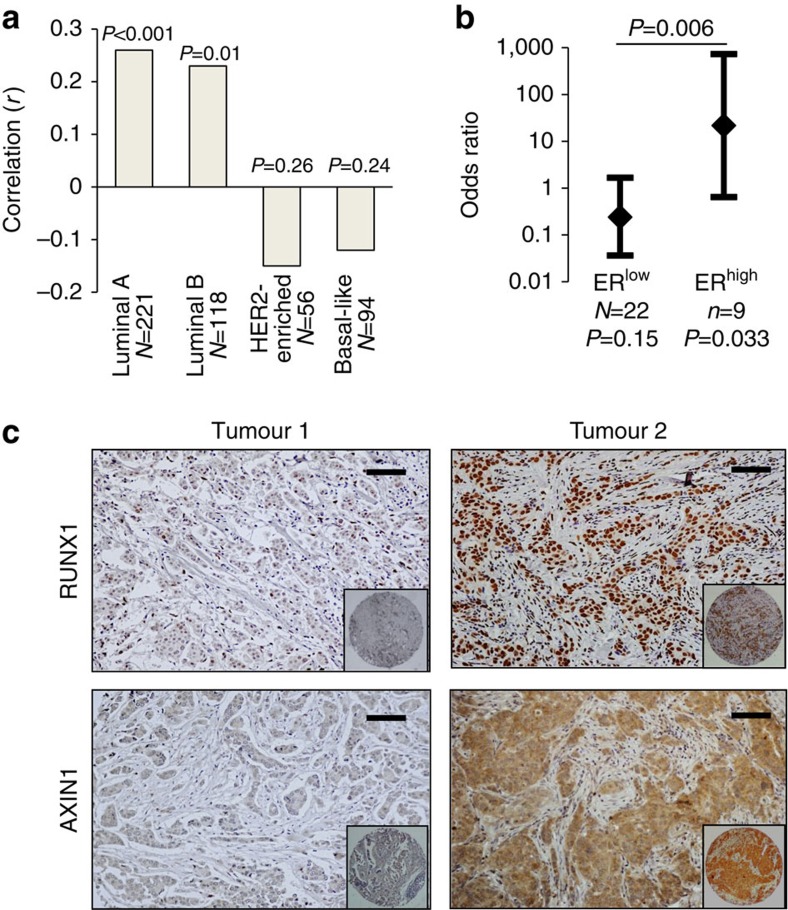
Association between RUNX1 and AXIN1 in ER^+^ breast cancer tumours. (**a**) Correlation between the RUNX1 inhibitory index and *AXIN1* mRNA in the breast cancer patient cohort of TCGA (ref. 20). The RUNX1 inhibitory index was calculated for each tumour as described in the ‘Methods' section and the correlation with *AXIN1* mRNA (UCSC isoform uc002cgp.1) was calculated. Bars represent the Pearson linear correlations (*r*) and *P* values were determined based on *r* and the sample size *N*. (**b**) Breast cancer tumour microarray TMA-1007 from Protein Biotechnologies, Inc. was immunostained for RUNX1 and AXIN1. The ER^+^ invasive ductal carcinomas were designated as positive or negative for RUNX1 and AXIN1. Data represent the odds ratio and the 95% confidence intervals for the association between AXIN1 status and RUNX1 status in tumours expressing ERα at either low (ER^low^) or high levels (ER^high^). Association between the RUNX1 status and AXIN1 status was tested using the Pearson chi-square test for the 2 × 2 table, for ER^low^ and ER^high^ tumours separately. Odds ratios for the ER^low^ and ER^high^ tumours were compared using the Breslow–Day test for homogeneity of odds ratios. (**c**) RUNX1 and AXIN1 immunohistochemical staining of two ER^high^ tumours from the TMA illustrating the association between RUNX1 and AXIN1 expression (Magnification × 20; scale bar, 50 μm; insets show the 1.1 mm cores in their entirety).

To address the combinatorial regulation of *AXIN1* by RUNX1 and oestrogens in clinical settings, we first calculated an ‘inhibitory index' for RUNX1 in each tumour in the breast cancer cohort of TCGA^20^ based on the expression levels of genes that RUNX1 inhibited in MCF7 cells (see ‘Methods' section). As shown in Fig. 5a, *AXIN1* mRNA significantly correlated with the RUNX1 inhibitory index in ER^+^ but not in the ER^−^ breast cancer types. We further analysed the association between AXIN1 and RUNX1 at the protein level using a commercial tumour microarray, TMA-1007, which included among others duplicate cores from 31 ER^+^ invasive ductal carcinomas in which ER was expressed at either low (ER^low^) or high levels (ER^high^). We immunostained the TMA to define each tumour as positive or negative for RUNX1 and AXIN1. Despite the reported scarcity of RUNX1 mutations (≤5% in three large independent studies^18^,^19^,^20^), RUNX1 was undetectable in 12 of the 31 ER^+^ ductal invasive carcinomas, indicating that its function may be lost, at least transiently, in far more than the ≤5% of tumours with *RUNX1* mutations (Supplementary Table 1). AXIN1 was undetectable in 11 of the 31 tumours, but there was no correlation between the RUNX1 and the AXIN1 status across all the tumours. However, examination of the correlation in relation to the ER levels demonstrated strong positive association between RUNX1 and AXIN1 (odds ratio of 21.7; *P*=0.033) in the ER^high^ tumours with no significant correlation in the ER^low^ tumours (Fig. 5b and Supplementary Table 1). The positive correlation in the ER^high^ group is illustrated by representative immunohistochemical images in Fig. 5c. Given the evidence from cell culture and animal models for combinatorial regulation of AXIN1 by RUNX1 and oestrogens (Fig. 4), the analyses of the RNA-seq and the TMA data (Fig. 5) suggest ER-dependent regulation of *AXIN1* by RUNX1 in patient tumours as well.

### Abbreviated mitosis in RUNX1-depleted breast cancer cells

**Figure 6:**
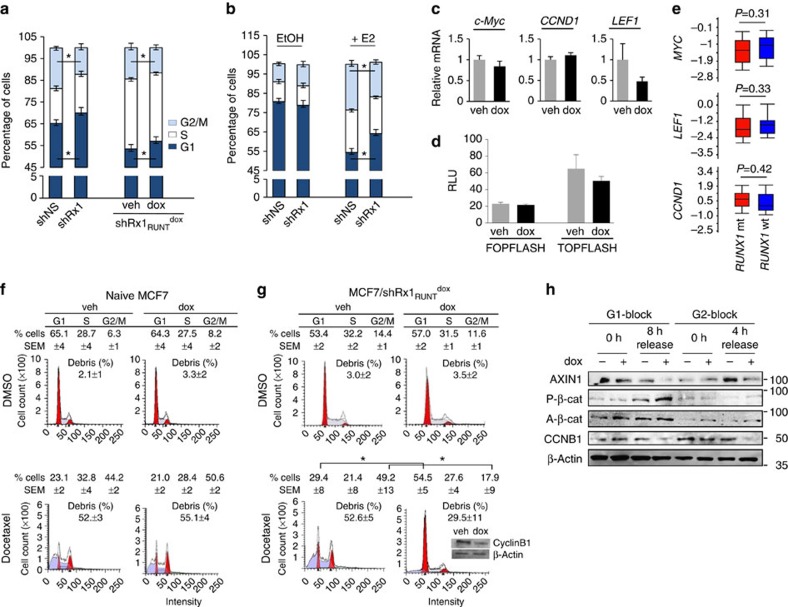
RUNX1 silencing deregulates breast cancer cell mitosis. RUNX1 was knocked down in MCF7 cells either constitutively (**a** (left) and **b**) or conditionally upon treatment with dox (**a** (right), **c**,**d g** and **h**). Cells were maintained in medium supplemented with either complete serum (**a**,**c**,**d**,**f**–**h**) or CSS (**b**). (**a**,**b**) Cell cycle profiles were obtained by FACS analysis of propidium iodide-stained cells. In **b**, cells were treated with either vehicle control (EtOH) or estradiol (E2) for 48 h as indicated. *P<0.05 by *t*-test. (**c**) RT–qPCR analysis of the indicated Wnt/cell cycle-regulatory genes. Data are corrected for 18S RNA. (**d**) Luciferase assay of TOPFLASH or control FOPFLASH as a measure of β-catenin/TCF activity. (**e**) Expression levels of the indicated genes in RUNX1-mt (*N*=17) versus RUNX1-wt (*N*=389) ER^+^ tumours in the breast cancer cohort of TCGA. Significance of the differences was calculated using Mann–Whitney test. (**f**,**g**) Naive MCF7 (**f**) and MCF7/shRx1_RUNT_^dox^ cells (**g**) were treated with 250 ng ml^−1^ dox for 72 h and 2 nM docetaxel was added for the last 48 h as indicated. Percentages of cells in G1, S and G2/M are given as mean and s.e.m. values from three independent experiments. Representative plots are presented, with inset in **g** showing western blot analysis of cyclin B1. (**h**) MCF7/Rx1sh_RUNT_^dox^ cells were synchronized as described in the ‘Methods' section at either G1/S or G2/M, or at the indicated time points. The cells were treated with 250 ng ml^−1^ dox along with the release from the first thymidine block and extracts were subjected to western blot analysis of the indicated proteins. Quantitative data are mean±s.e.m. from three independent experiments. *P<0.05 by *t*-test.

Activation of the Wnt/β-Catenin pathway is traditionally thought to promote cell proliferation through LEF/TCF-mediated stimulation of target genes such as *CCND1*, *c-MYC* and *LEF1* itself, resulting in accelerated G1/S cell cycle transition^39^,^49^,^50^. However, cell cycle profiling showed that RUNX1 silencing, either constitutively or conditionally (by dox treatment), resulted in increased, not decreased percentage of cells in the G1 phase of the cell cycle (Fig. 6a,b). Accordingly, *RUNX1* silencing increased the mRNA levels of neither *c-Myc*, nor *CCND1* nor *LEF1* (Fig. 6c and Supplementary Fig. 5), nor did it upregulate expression of the LEF/TCF TOPFLASH reporter (Fig. 6d). Furthermore, expression of neither *c-Myc*, *CCND1*, nor *LEF1* differed in TCGA breast cancer tumours with wild-type versus mutant *RUNX1* (Fig. 6e). Rather than accelerated G1/S transition, the cell cycle profiles (Fig. 6a,b) suggested abbreviated mitosis, indicated by a reproducible decrease in G2/M cells, and this effect was observed with both complete (oestrogen-containing) medium (Fig. 6a) and after the supplementation of CSS with E2 (Fig. 6b).

To further investigate the role of RUNX1 in regulating breast cancer cell cycle progression, we challenged control and RUNX1-depleted MCF7 cells by treatment with the anti-mitotic taxane drug docetaxel, which normally induces a G2/M block and a mitotic catastrophe^51^,^52^. We first confirmed (Fig. 6f) the accumulation of cells in G2/M, as well as the presence of cell debris, in naive cultures treated with 2 nM docetaxel. A similar response was observed in MCF7/RUNX1_RUNT_^dox^ cells cultured in the absence of dox as control (Fig. 6g, left). However, dox-treated (RUNX1-depleted) MCF7/RUNX1_RUNT_^dox^ cells (Fig. 6g, right), and not dox-treated naive MCF7 cells used as control (Fig. 6f, right) were resistant to docetaxel. Consistent with abbreviated mitosis, cyclin B1 levels decreased in the RUNX1-depleted, docetaxel-treated compared with control cells (Fig. 6g, inset). Similar results were obtained with the microtubule-targeting agent nocodazole (Supplementary Fig. 6).

Mitotic abbreviation in RUNX1-depleted ER^+^ breast cancer cells could result from deregulation of β-catenin at the centrosome, where it is locally phosphorylated and controls microtubule organization before and during mitosis^33^,^34^,^36^. Given its cell cycle-dependent expression^53^ (Supplementary Fig. 7), we measured phospho-β-catenin (P-β-cat) levels by western blot analysis of cells blocked at G1/S, G2/M or after release. As shown in Fig. 6h, RUNX1 depletion resulted in increased P-β-cat levels specifically around 8 h after release from a G1/S block. The cell cycle-dependent increase in P-β-cat, which is undetectable in unsynchronized cells (Supplementary Fig. 8) may account for deregulated mitosis in RUNX1-depleted ER^+^ breast cancer cells through its role in the centrosome^33^,^34^,^35^,^36^ (Supplementary Fig. 9).

### Partial restoration of cell cycle control by AXIN1 stabilization

**Figure 7:**
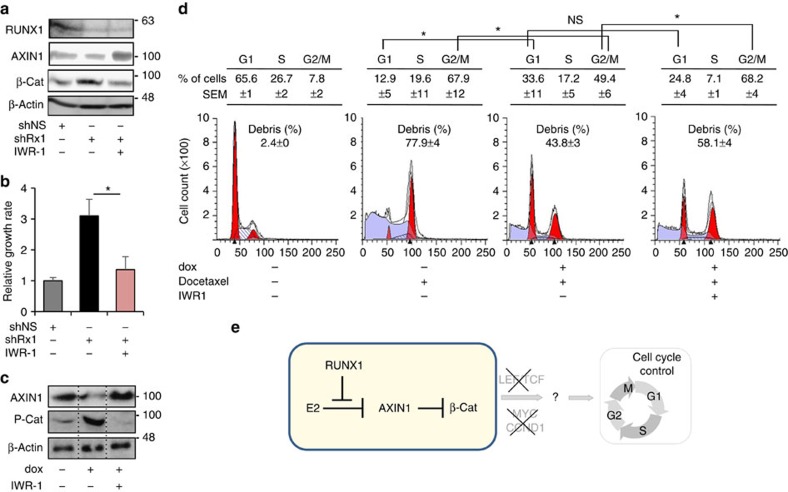
AXIN1 stabilization normalizes β-catenin and partially restores cell cycle control in RUNX1-depleted cells. (**a**) MCF7 cells constitutively expressing shRx1_3′-UTR_ (shRx1) were treated for 36 h with either 5 μM IWR1 or its dimethyl sulphoxide vehicle followed by western blot analysis of the indicated proteins. MCF7 expressing a nonspecific shRNA (shNS) were analysed as a reference control. (**b**) Cells as in **a** were treated as indicated for 6 days and their growth rate was calculated based on MTT assays as in Fig. 2b. *P<0.05 by *t*-test. (**c**) AXIN1 and P-β-cat levels were assessed 8 h after the release of MCF7/shRx1_RUNT_^dox^ cells from a G1/S double thymidine block as in Fig. 6h. Dox treatment (to silence RUNX1) initiated along with the release from the first thymidine block and IWR1 treatment (to stabilize AXIN1) initiated 17 h before harvest. (**d**) MCF7/shRx1_RUNT_^dox^ cells were treated for 72 h with dox (to silence RUNX1) and 2 nM docetaxel for 48 h (to induce mitotic slippage) as in Fig. 6f,g and IWR1 was added for the last 24 h before FACS analysis. Data are mean±s.e.m. (*n*=3). *P<0.05 by *t*-test. (**e**) Working model for the tumour suppressor function of RUNX1 in ER^+^ breast cancer, whereby RUNX1 prevents E2-mediated *AXIN1* suppression. Mechanisms linking the RUNX1/AXIN1/β–catenin axis to loss of cell cycle control in RUNX1-deficient ER^+^ mammary epithelial cells remain to be fully elucidated. They entail stimulation of neither LEF/TCF, nor *c-MYC*, nor *CCND1,* nor G1/S phase transition, but are associated instead with deregulated mitosis.

Because RUNX1 loss resulted in decreased AXIN1, the least abundant component of the β-catenin destruction complex and a rate-limiting factor for β-catenin phosphorylation and degradation^32^,^54^, we explored the effects the AXIN1 stabilizer IWR1 (ref. 55) on the deregulated cell cycle in RUNX1-depleted MCF7 cells. Remarkably, the IWR1-mediated restoration of AXIN1 and the subsequent normalization of β-catenin levels in MCF7/shRx1 cells (Fig. 7a) resulted in a decrease in cell growth rate (Fig. 7b) to levels measured in control MCF7/shNS cultures. Furthermore, IWR1 prevented the cell cycle-dependent increase in P-β-cat level (Fig. 7c) and restored docetaxel-mediated G2/M block (Fig. 7d) in dox-treated (RUNX1-depleted) MCF7/shRx1^dox^ cells. Taken together, our results assign a role for RUNX1 in antagonizing oestrogen-mediated AXIN1 suppression and highlight AXIN1 as a potential target for the treatment of RUNX1-deficient ER^+^ breast cancer (Fig. 7e).

## Discussion

This study demonstrates combinatorial regulation of *AXIN1* by RUNX1 and oestrogen signalling in ER^+^ breast cancer cells. *AXIN1* mRNA and protein levels were decreased upon RUNX1 silencing, and this was observed only in the presence of oestrogen. It did not occur in (1) ER^−^ breast epithelial cell lines (Fig. 4d); (2) ER^+^ breast cancer cells treated with the ICI 182780 (Fig. 4b); and (3) ER^+^ breast cancer cells cultured in CSS without added E2 (Fig. 4a). That RUNX1 regulates *AXIN1* in an oestrogen-dependent manner *in vivo* is suggested by (1) the decreased *Axin1* expression in the predominantly ER^+^ ML cells, but not in the predominantly ER^−^ LP cells in Runx1-deficient versus control murine mammary epithelium (Fig. 4e), (2) the positive correlation between AXIN1 mRNA and the RUNX1 inhibitory index in ER^+^ but not ER^−^ breast cancer subtypes in TCGA (Fig. 5a) and (3) the positive correlation between RUNX1 and AXIN1 protein levels in ER^high^ but not ER^low^ human ER^+^ breast cancer tumours (Fig. 5b). Importantly, RUNX1 did not stimulate *AXIN1* expression in the absence of oestrogens; rather, it prevented oestrogen-mediated *AXIN1* repression (Fig. 4). Such combinatorial regulation by RUNX1 and oestrogens does not represent genome-wide antagonism of oestrogen-regulated gene expression (Fig. 4c); instead, it likely reflects local interaction between ERα and RUNX1 at a co-occupied regulatory region in the second intron of *AXIN1* (Fig. 3c). This co-occupancy does not seem to occur through a mechanism of tethering as described for forcibly expressed ERα in ER^−^ MDA-MB-231 cells^29^. Evidence arguing against recruitment of endogenous ERα by DNA-bound RUNX1 in MCF7 cells has been presented previously^29^ and is certainly unlikely at the *AXIN1* locus, where the ERα and RUNX1 ChIP-seq peaks are phased, each perfectly aligned with its respective DNA sequence motif (Fig. 3c). Although genome-wide transcriptional regulation by RUNX1 is not generally dependent on oestrogens (Fig. 4c, Supplementary Fig. 3), such dependence at a few critical regulatory loci, such as *AXIN1*, may explain the specificity of RUNX1 recurrent mutations to ER^+^ breast cancer tumours^18^,^20^. While additional loci (for example, *NCRNA00173*, *LGR6*, *TFF3* and *CBFA2T3*, *MMP17*; Fig. 3b) may also contribute to the oestrogen-dependent tumour suppressor activity of RUNX1 in breast cancer, the role of *AXIN1* downstream of RUNX1 in this context is strongly supported by deregulation of β-catenin in RUNX1-deficient ER^+^ breast cancer cells and the corrective effects of IWR1. Our data therefore demonstrates crosstalk between oestrogen and β-catenin signalling in ER^+^ breast cancer through AXIN1. It further suggests that RUNX1 suppresses ER^+^ breast cancer progression by denying oestrogens their negative regulation of *AXIN1*.

Our work begins to elucidate tumour suppression mechanisms operative downstream of the RUNX1-AXIN1 axis in ER^+^ breast cancer cells. As expected, loss of *AXIN1* expression after RUNX1 silencing in the MCF7 and T47D cell lines was associated with increased β-catenin levels (Fig 1d,e). However, unlike colon cancer, increased β-catenin in breast cancer does not seem to deregulate the β-catenin/TCF-driven transcription in the canonical Wnt pathway^37^,^38^. Accordingly, the upregulation of β-catenin in RUNX1-depleted ER^+^ breast cancer cell lines was not associated with increased expression of the LEF/TCF-responsive G1/S regulatory genes *CCND1* and *c-Myc* (Fig. 6c; Supplementary Fig. 5). Likewise, the Wnt reporter TOPFLASH was not stimulated (Fig. 6d) and G1/S cell cycle transition was not accelerated (Fig. 6a,b). Instead, E2-mediated *AXIN1* suppression upon RUNX1 loss may contribute to ER^+^ breast cancer progression through mitotic aberrations that promote expansion of a stem cell-like population. This notion is supported by the following observations: (1) accelerated growth and upregulation of stem cell markers in RUNX1-depleted ER^+^ breast cancer cells (Fig. 2a–e); (2) increased *Sox2* mRNA levels in *Runx1*-ablated murine mammary epithelial cells and *RUNX1*-mutant human breast cancer tumour biopsies (Fig. 2f,g); (3) cell cycle-dependent upregulation of P-β-cat in RUNX1-depleted MCF7 cells (Fig. 6h), which could potentially affect centrosome-anchored microtubule asters and mitotic cell polarity^34^,^56^,^57^; (4) loss of cyclin B1 in RUNX1-depleted MCF7 cells (Fig. 6g), potentially reflecting deregulated microtubule organization and premature activation of the anaphase-promoting complex^58^; and (5) abbreviated mitosis/slippage in RUNX1-depleted MCF7 breast cancer cells (Fig. 6a,g). Thus, the increased levels of SOX2, AXIN2 and CD44 (Fig. 2c) do not appear to reflect deregulated LEF/TCF-driven transcription. Instead, they likely represent one aspect of a stem cell-like phenotype, potentially related to changes in centrosomal proteins including β-catenin and possibly RUNX1 itself^59^.

The consequences of β-catenin stimulation in RUNX1-depleted ER^+^ luminal cells remain to be fully elucidated. They clearly differ, however, from mechanisms activated by stimulation of the Wnt/β-catenin pathway in mouse models where MMTV-driven genetic manipulations lead to breast carcinogenesis^38^,^60^,^61^,^62^. Such genetic manipulations typically result in the development of basal and alveolar ER^-^ tumours through mechanisms resembling Wnt-driven colon carcinogenesis^60^,^61^,^62^. Possibly, the ER^+^ luminal cell population is spared in these models because it lacks Wnt/β-catenin-responsive stem cells^46^. Our study suggests that these same ER^+^ cells specifically respond to loss of RUNX1 function by E2-dependent downregulation of AXIN1, and that the mechanisms operative downstream of the RUNX1/AXIN1/β-catenin axis in these cells are distinct from those operative in Wnt-driven colon cancer and ER^−^ breast cancer.

Further studies are also warranted to investigate the role of RUNX1 in the control of mammary epithelial cell physiology. We speculate that RUNX1 may prevent undesirable β-catenin-driven stem cell proliferation in adult ER^+^ luminal cells by antagonizing ER that is activated by circulating oestrogenic compounds and binds at the *AXIN1* locus; and, downregulation of RUNX1, as well as RUNX2 and RUNX3, during pregnancy and lactation, and their upregulation during involution^13^,^63^ may facilitate physiological regulation of β-catenin in response to hormonal changes during these processes.

Compromised RUNX1 function in ER^+^ breast cancer likely occurs in far more than the <5% of tumours with *RUNX1* mutations^20^. RUNX1 expression may be compromised in many additional cases, including the 20–40% invasive ductal carcinomas in which RUNX1 is undetectable by immunohistochemistry (Supplementary Table 1 and refs 12, 15). Compromised RUNX1 transcription may be related to promoter hypermethylation (Supplementary Fig. 2), and protein expression and function may also be lost posttranslationally, for example, due to *CBFB* mutations^18^,^19^,^20^. Subsequent loss of AXIN1 in RUNX1-deficient ER^+^ breast cancer cells may be prevented by treatment with tankyrase inhibitors^64^,^65^ (Fig. 7), partly alleviating consequences of RUNX1 loss. Tankyrase inhibition would likely be safer than the alternative of restoring RUNX1 itself because RUNX proteins play both tumour suppressor and oncogenic roles in cancer^1^,^2^. In fact, RUNX1 appears to play an oncogenic rather than a tumour suppressor role in triple-negative breast cancer^12^,^15^, possibly, in part, related to alternative splicing of *AXIN1* (ref. 66). In ER^+^ breast cancer, however, *RUNX1* predominantly functions as a tumour suppressor, and the present work attributes this function at least in part to antagonism of oestrogen-mediated *AXIN1* suppression.

## Methods

### Clinical data mining

High-throughput whole-genome data used in this study was from cases for which clinical information was available in the breast cancer cohort of TCGA^20^. Gene expression (based on either microarray hybridization or RNA-seq), DNA methylation and somatic mutation data were retrieved from the TCGA Data Portal ( http://cancergenome.nih.gov/). In addition, RNA-seq data were downloaded from the UCSC Cancer Genomics Browser ( http://genome-cancer.ucsc.edu) as ranked expression scores. Expression microarray data and *RUNX1* mutation status of 209 ER^+^ tumours described by Ellis *et al.*^18^ was obtained from the University of North Carolina Microarray Database.

### Mice

Mice used in this study have been previously described^13^. YFP^+^ ER^−^ luminal progenitor cells (LPs) were sorted from *MMTV-Cre;Runx1*^*f/f*^*;R26Y* and *MMTV-Cre;Runx1*^*+/+*^*;R26Y* (control) females (2 months of age) and YFP^+^ ER^+^ mature luminal cells (MLs) were sorted from *MMTV-Cre;Runx1*^*f/f*^*;Rb1*^*f/f*^*;R26Y* and *MMTV-Cre;Runx1*^*+/+*^*;Rb1*^*f/f*^*;R26Y* (control) females (either 2-month or 7-month old). *R26Y* is a conditional Cre-reporter that expresses YFP upon Cre-mediated recombination. In the compound mice, conditional knockout of *Runx1* (and *Rb1*) in mammary epithelial cells is linked to YFP expression. Breeding with *Rb1*^*f/f*^ mice facilitated rescue of the ER^+^ luminal cell subpopulation upon *Rb1* deletion as described^13^. FACS sorting was performed with a FACSAria sorter (BD Biosciences) using antibodies from eBiosciences including CD24-eFluor450, CD24-eFluor605, CD29-APC, c-Kit-PE-CY7, CD14-PE and biotinylated CD31, CD45, TER119 and Streptavidin-PerCP-CY5.5. All animal work was performed in strict accordance with the recommendations in the Guide for the Care and Use of Laboratory Animals of the National Institutes of Health and was approved by the Institutional Animal Care and Use Committee of Boston Children's Hospital where the animals are housed.

### Cells

The ER^+^ MCF7 and T47D, as well as the ER^−^ MDA-MB-231 breast cancer cells were from the American Type Culture Collection. MCF10A cells were obtained from the Karmanos Cancer Institute (Detroit, Michigan). MCF7 and T47D cells were cultured in Dulbecco's Modified Eagle's medium (DMEM; Mediatech, Inc) and RPMI-1640 medium (Mediatech, Inc), respectively, both supplemented with 10% fetal bovine serum (FBS; Gemini Bio-products). MDA-MB-231 cells were cultured in DMEM/F12 (Mediatech, Inc) supplemented with 5% FBS. MCF10A cells were cultured in DMEM/F12 supplemented with 5% horse serum (Gemini Bio-products), 10 μg ml^−1^ insulin (Sigma-Aldrich), 20 ng ml^−1^ EGF (Sigma-Aldrich), 0.5 μg ml^−1^ hydrocortisone (Sigma-Aldrich) and 0.1 M CaCl_2_. For oestrogen treatment, the cells were washed three times with phosphate-buffered saline and maintained for 48 h in phenol-red-free growth medium supplemented with 10% CSS (Gemini Bio-products), followed by estradiol (E2) administration (Sigma-Aldrich). ICI 182780, IWR1 and docetaxel, also from Sigma-Aldrich, were added to the culture medium as indicated.

### *In vitro* RUNX1 manipulation

Mission shRNA lentiviral plasmids targeting either the RUNT or the 3′-UTR of RUNX1 were purchased from Sigma-Aldrich (Supplementary Table 2). For packaging, the plasmids were co-transfected into HEK293T cells along with helper plasmids pMD.G1 and pCMV.R8.91 using the calcium chloride method. Culture media containing viral particles were harvested after 48–72 h and used for the transduction of breast cancer cells in the presence of 8 μg ml^−1^ polybrene (Millipore Corp., MA, USA) followed by selection with 1 μg ml^−1^ puromycin for 5 days. Alternatively, we used the pSLIK vector system^67^ for conditional RUNX1 silencing. Lentiviruses containing dox-inducible shRNAs that target either the RUNT domain (shRx1_RUNT_^dox^) or the 3′-UTR (shRx1_3′-UTR_^dox^) of RUNX1 (Supplementary Table 2) were engineered by first cloning the shRNAs into the entry vector pEN_TmiRc3. The resulting plasmids were each recombined using Gateway LR Clonase II enzyme mix (Invitrogen) with the pSLIK destination vector carrying a neomycin-resistant gene. Transduced cells were selected with Geneticin (Gemini Bio-products) at 1 mg ml^−1^ for MCF7 cells and 0.4 mg ml^−1^ for T47D cells. The pSLIK vector system was also used to conditionally express FLAG-tagged RUNX1, either wild type or mutant, by following the protocol previously described for conditional expression of FLAG-tagged RUNX2 (refs 9, 68). For RUNX1 rescue experiments, cells containing dox-inducible FLAG-RUNX1 (Rx1^dox^) were additionally transduced with a lentivirus expressing the Mission shRNA against the 3′-UTR of RUNX1.

### Cell proliferation and cell cycle assays

Cell proliferation was assessed using 3-(4,5-dimethylthiazol-2-yl)-2,5-diphenyltetrazolium bromide (MTT) colorimetric assay. Approximately 5,000 cells per well were seeded in 96-well tissue culture plates and incubated on the indicated days with 0.5 mg ml^−1^ MTT (Sigma-Aldrich) for 3 h before lysis with dimethyl sulphoxide. Absorbance at a 595 nm wavelength was measured using Victor_3_V plate reader from PerkinElmer. Cell cycle synchronization at G1/S was achieved by double-thymidine block. The cells were incubated for 16 h in medium containing 2 mM thymidine (Sigma-Aldrich), released for 8–12 h in DMEM containing 10% FBS, and then incubated again in 2 mM thymidine for 16–18 h. For G2/M synchronization, the cells were released from the double-thymidine block for 4 h before treatment with 100 nM nocodazole (Sigma-Aldrich) for 14 h. Percentage of cells in the various phases of the cell cycle was quantified by propidium iodide staining and flow cytometry using LSRII flow cytometer (BD Biosciences) and the Modfit LT SynchWizard Tool (Verity Software House).

### TOPFLASH assay

The cells were seeded at a density of 50,000 per well in 24-well plates, and LEF/TCF-mediated transcriptional activity was measured using the Super8xTOPFLASH reporter plasmid with the Super8xFOPFLASH plasmid serving as control. Plasmids were transiently transfected using the jetPrime transfection reagent (Polyplus-Transfection) according to the manufacturer's specification. Luciferase activity was determined with the Dual-Luciferase Reporter Assay System (Promega) using a Victor_3_V plate reader (PerkinElmer).

### RT–qPCR

Total RNA from cultured cells was isolated using Aurum Total RNA mini-kit (Bio-Rad) and cDNA was synthesized from 1 μg of total RNA with iScript reverse transcription kit (Bio-Rad). Quantitative real-time PCR was carried out in triplicate using a CFX96 instrument (Bio-Rad) and the Maxima SYBR Green/Fluorescein Master Mix (ThermoFisher Scientific). Relative mRNA expression values were normalized to those of GAPDH and/or 18S RNA. For cell populations isolated from mouse tissue, the RNA was extracted using RNeasy kit (Qiagen) and amplified with the Ovation RNA Amplification System V2 kit (Nugen). cDNA was synthesized with Omniscript (Qiagen) according to the manufacturer's protocol, and real-time qPCR was performed using FastStart SYBR Green Master (Roche). Gene expression in ER^-^ LPs was measured in LPs sorted from *MMTV-Cre;Runx1*^*f/f*^*;R26Y* and *MMTV-Cre;Runx1*^*+/+*^*;R26Y* (control) adult females. Since the ER^+^ ML subpopulation was largely lost in *MMTV-Cre;Runx1*^*f/f*^*;R26Y* females^13^, gene expression in ER^+^ MLs was measured in rescued MLs (by simultaneous inactivation of *Rb1* together with *Runx1* (ref. 13) sorted from *MMTV-Cre;Runx1*^*f/f*^*;Rb1*^*f/f*^*;R26Y* female mice (7 months of age), upon normalization to those in ER^+^ MLs sorted from 2-month-old *MMTV-Cre;Runx1*^*f/f*^*;Rb1*^*f/f*^*;R26Y* female mice (to correct for changes in cell compositions in the ML FACS gate) and to those in the corresponding ML subpopulations sorted from 7-month- and 2-month-old *MMTV-Cre;Runx1*^*+/+*^*;Rb1*^*f/f*^*;R26Y* control females, respectively. Primers used for RT–qPCR amplifications are listed in Supplementary Table 2.

### Western blot analysis

The cells were lysed using RIPA buffer (ThermoFisher Scientific) supplemented with protease (Sigma-Aldrich) and phosphatase inhibitors (ThermoFisher Scientific). Cytoplasmic and nuclear extracts were prepared using NE-PER kit (ThermoFisher Scientific) following the manufacturer's instructions. Proteins were resolved by SDS–PAGE (polyacrylamide gel electrophoresis) and transferred to AmershamHybond-P PVDF membranes (GE Healthcare), followed by blocking with 5% non-fat dry milk. The following antibodies were purchased from Cell Signaling Technology, Inc. and used at a 1:1,000 dilution: rabbit anti-RUNX1 (#8529), rabbit-anti-AXIN1 (#2087), rabbit anti-phospho-β-catenin (Ser33/37/Thr41; #9561) and mouse anti-CCNB1 (#4135). Mouse anti-active-β-catenin (#05-665) was purchased from Millipore and used at a 1:1,000 dilution. Rabbit anti-β-catenin (ab32572) and rabbit anti-SOX2 from Abcam were used at a 1:5,000 dilution. Mouse anti-FLAG (F3165) antibody from Sigma-Aldrich was used at a 1:2,000 dilution. Goat anti-Actin (sc-1616) and goat anti-GAPDH (sc-20357) antibodies were purchased from Santa Cruz Biotechnology and used at a 1:200 dilutions. The mouse monoclonal anti-β-tubulin antibody, developed by Dr Charles Walsh, was obtained from the Developmental Studies Hybridoma Bank under the auspices of the NICHD and The University of Iowa, Department of Biological Sciences, Iowa City, USA, and used at a 1:5,000 dilution. HRP-conjugated donkey anti-goat (sc-2033), donkey anti-mouse (sc-2314) and goat anti-rabbit (sc-2004) antibodies were purchased from Santa Cruz Biotechnology and used at a 1:2,000 dilution. Immunodetection was performed using Pierce ECL2 western blotting detection system (ThermoFisher Scientific). Uncropped scans of key western blots are provided as a Supplementary Fig. 10.

### Immunohistochemistry

TMA slides (TM-1007) were purchased from Protein Biotechnologies, Inc. The majority of tumours represented on this TMA were invasive ductal carcinomas, of which 31 were ER^+^ and 33 were ER^−^. TMAs were deparaffinized and rehydrated before antigen retrieval in 10 mM sodium citrate buffer at 95 °C. After cool-down, the slides were incubated for 20 min in 1% H_2_O_2_ in methanol to block endogenous peroxidase, washed with TBS and blocked with Background Punisher (#BP974, Biocare Medical) for 10 min at room temperature. The slides were then incubated overnight at 4 °C with antibodies against RUNX1 (#8529) or AXIN1 (#2087), both purchased from Cell Signaling Technology and used at a 1:50 dilution. Afterwards, the slides were washed with TBS, and treated with MACH4 Universal HRP-polymer (#M4U534, Biocare Medical) for 30 min. After 5-min incubation with a DAB reagent (#K3466, DAKO) to visualize HRP, the slides were rinsed, counterstained with haematoxylin and topped with cover slips. ER histoscores were provided by the manufacturer and presence or absence of RUNX1 and AXIN1 was determined by a certified surgical pathologist (P.M.-F.). The association between the RUNX1 status and AXIN1 status was tested using the Pearson chi-square test for the 2 × 2 table, for ER^low^ and ER^high^ tumours separately. Odds ratios for the ER^low^ and ER^high^ tumours were compared using the Breslow–Day test for homogeneity of odds ratios.

### Expression profiling and ChIP-seq

MCF7/shRx1 and MCF7/shNS cells, expressing shRNAs that target RUNX1 or a nonspecific shRNA, respectively, were maintained in CSS for 48 h before treatment with either 10 nM E2 or the ethanol vehicle for 48 h. Total RNA was extracted using Aurum Total RNA mini-kit (Bio-Rad) and global expression profiling was performed using BeadChips (Illumina) by the Southern California Genotyping Consortium at UCLA. To compare the RUNX1 and the RUNX2 transcriptomes, MCF7 cells were first transduced with the respective dox-inducible pSLIK vectors, and then with the Mission shRx1_3′-UTR_ vector. RUNX1 depleted cells were then treated with 0.25 μg ml^−1^ dox to induce RUNX1 or RUNX2 expression and/or with 10 nM E2 for 48 h, and global mRNA expression was profiled as described above. RUNX1 ChIP-seq was performed essentially as described^69^. Briefly, MCF7 cells were cross-linked, lysed and sonicated to obtain DNA fragments mostly in the 200–1,000-bp range. Immunoprecipitation was performed at 4 °C overnight with anti-RUNX1 antibody (ab23980, Abcam). Downstream analysis was performed on high-fidelity peaks reproduced in two independent experiments. GEO accession numbers for the microarray and ChIP-Seq data are GSE65620, GSE65616 and GSE65313. MCF7 ERα ChIP-seq data was from GSE14664. ChIP-qPCR validation was performed using antibodies against either RUNX1 (ab23980, Abcam) or ERα (sc543x from Santa Cruz Biotechnology). For ERα, ChIP-qPCR cells were maintained in CSS for 48 h and treated with 10 nM E2 for 1 h. A region near *GAPDH* served as negative control.

### Data analysis

Global gene expression raw data from MCF7 cell cultures was processed using GenomeStudio (Illumina Inc). After background subtraction and quantile normalization, the signal intensity values were exported to the Partek Genomics Suite 6.4 (Partek, Inc.) using ‘Partek's Report Plug-in'option in the GenomeStudio software and differential expression was analysed by one-way analysis of variance. Differentially expressed genes were investigated using the Ingenuity Pathways Analysis package ( http://www.ingenuity.com). Fisher's exact test as implemented in the Ingenuity Pathways Analysis software was used to calculate *P* values. To estimate RUNX1 inhibitory activity on the basis of RNA-seq tumour data mining, we first defined a set of 123 genes that were expressed in E2-treated MCF7/shRx1_3′-UTR_ cells at levels ≥1.7-fold higher (*P*<0.05) than the respective expression levels in MCF7/shNS cells (Fig. 3a). The ranked expression score for each of these gene in each tumour in the breast cancer cohort of TCGA was obtained from the UCSC Cancer Genomics Browser. We then defined an ‘inhibitory index' for RUNX1 in each tumour as the mean rank for the 123 genes and calculated its correlation with the respective *AXIN1* expression level. For the analysis of RUNX1 locations, the ChIP-seq reads from biological replicates and an input control library were aligned to the hg19 build of the human genome. Peaks for RUNX1, as well as for ERα (based on Welboren *et al.*^47^) were called with MACS^70^ with default parameters using input as control and only reproducible peaks in both RUNX1 replicates were retained for further analysis. Unless otherwise stated, statistical significance of differences in this study was determined using InStat 3.0b GraphPad Software (GraphPad Software, Inc., CA, USA).

## Additional information

**Accession codes:** Microarray and ChIP-Seq data have been deposited in the GEO under accession codes GSE65620, GSE65616 and GSE65313.

**How to cite this article:** Chimge, N. *et al.* RUNX1 prevents oestrogen-mediated AXIN1 suppression and β-catenin activation in ER-positive breast cancer. *Nat. Commun.* 7:10751 doi: 10.1038/ncomms10751 (2016).

## Supplementary Material

Supplementary Information Supplementary Figures 1-10 and Supplementary Tables 1-2

Supplementary Data 1 Genome wide expression microarray (Agilent) and somatic mutation data of breast invasive ductal carcinoma was downloaded from the TCGA Data portal (https://tcga-data.nci.nih.gov/tcga/). Level 3 somatic mutation data was used to determine RUNX1 mutation status of tumors and differential gene expression (Fold Change>1.2 p<0.05) was determined using ANOVA as implemented in Partek Genomics Suite 6.4. Positive and negative fold-change values represent up- or down-regulation in the 17 RUNX1 mutant tumors versus 389 RUNX1 ER-positive tumors without RUNX1 mutations. p values represent the statistical significance of the changes by ANOVA.

Supplementary Data 2 Genome wide expression microarray data and RUNX1 mutation status of the respective breast tumors in the cohort of Ellis et.al., 2012 (PMID: was provided from the University of North Carolina Microarray Database and differential gene expression (Fold Change>1.5 p<0.05) was determined using ANOVA as implemented in Partek Genomics Suite 6.4. Positive and negative Fold-change values represent up- or down-regulation in the 7 RUNX1 mutant tumors versus 202 ER-positive tumors without RUNX1 mutations. p values represent the statistical significance of the changes by ANOVA.

Supplementary Data 3 The genes differentially expressed in ER-positive RUNX1-mutant tumors versus ER-positive tumors without RUNX1 mutations in both TCGA and the cohort of Ellis et al. and listed in Supplemental Data 1 and the Supplemental Data 2 respectively, were interrogated for common genes using venn diagram function in Partek Genomics Suite 6.4.

Supplementary Data 4 RUNX1 was knocked down in MCF7 cells using shRNAs targeting 3'UTR region of RUNX1. Gene expression profiling was generated on the Illumina HumanHT-12 v4 Expression Beadchip array. Raw data processing was performed using GenomeStudio (Illumina Inc). After background subtraction and quantile normalization the signal intensity values were exported to the Partek Genomics Suite{trade mark, serif} 6.4 (Partek, Inc.) using “Partek's Report Plug-in” option in the GenomeStudio software and differential expression was analyzed by one-way ANOVA. Genes differentially expressed in shRx1 expressing cells versus cells expressing non-specific short hairpin RNA (shNS) were analysed both in the absence and presence of estradiol.
