# Supplementary material for: RUNX1 prevents oestrogen-mediated AXIN1 suppression and β-catenin activation in ER-positive breast cancer
Source: Nat Commun. 2016 Feb 26;7:10751. doi: 10.1038/ncomms10751 (PMC4773428; doi:10.1038/ncomms10751)
Supplement: Supplementary Information — Supplementary Figures 1-10 and Supplementary Tables 1-2 [file ncomms10751-s1.pdf]

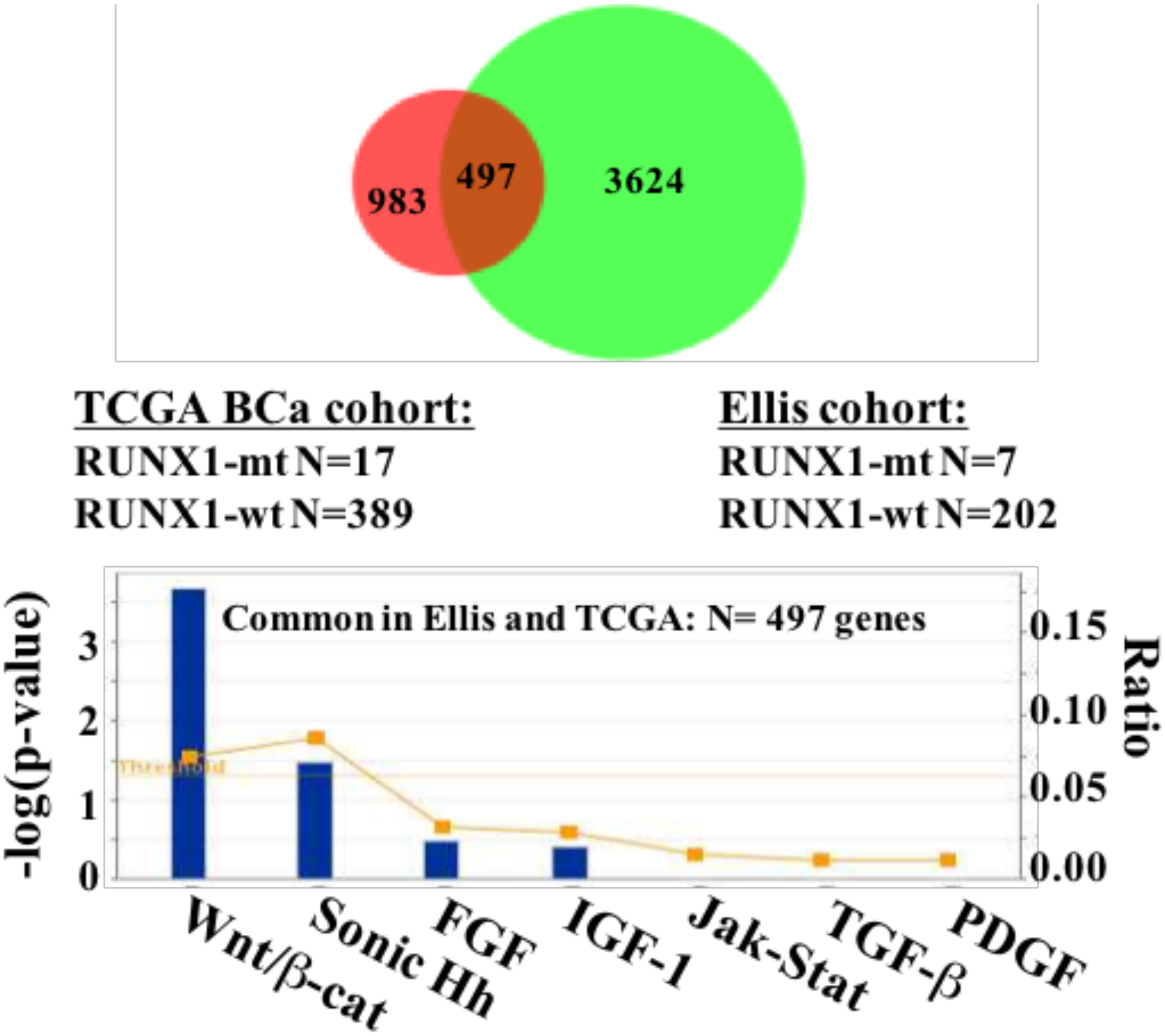


**Supplementary Fig. 1. Annotations of genes differentially expressed in breast cancer tumors with *RUNX1* mutations in two independent cohorts suggest deregulation of Wnt/β-catenin signaling.**

Genes differentially expressed in $ER^+$ tumors with wild-type versus mutant *RUNX1* in the breast cancer cohorts of both TCGA (https://tcga-data.nci.nih.gov/tcga/) and Ellis et al. (PMID: 22722193), listed in Supplemental Data 3, were interrogated by IPA™ for annotations related to major developmental signaling pathways. Line graph represent fold enrichment and bars represent statistical significance calculated by Fisher's exact test as implemented in the IPA™ software.

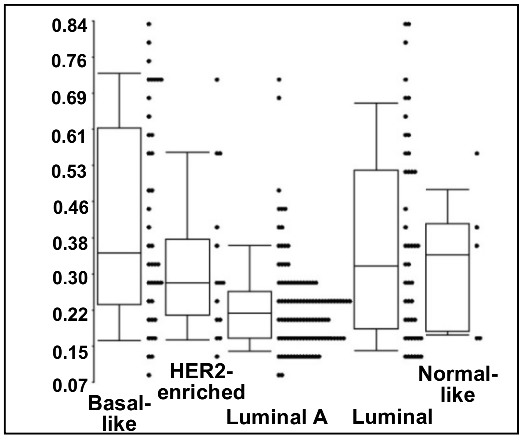


**Supplementary Fig. 2.** ***RUNX1*** **methylation across breast cancer subtypes**
*RUNX1* methylation status in the five major breast cancer subtypes was assessed based on data for probe cg01265860 retrieved from the breast invasive carcinoma cohort of TCGA (https://tcga-data.nci.nih.gov/tcga/). Methylation levels differ significantly between subtypes (p=1.96e-08 by ANOVA).
.

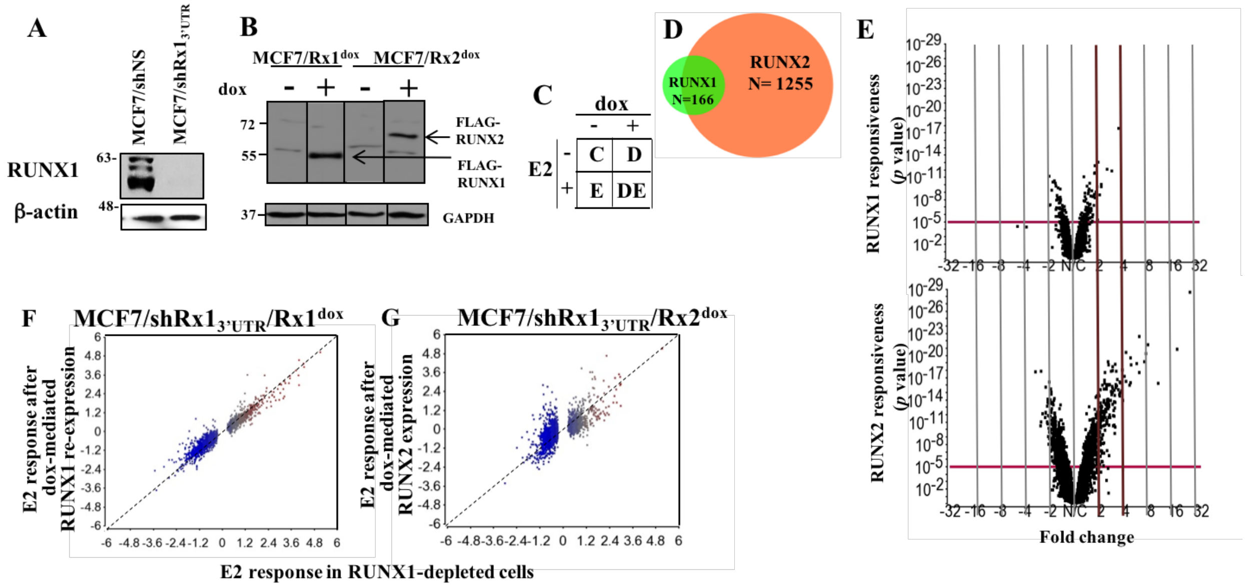


**Supplementary Fig. 3. RUNX1 vs. RUNX2 transcriptome in MCF7 cells.**
MCF7 cells were engineered to silence RUNX1 constitutively and express FLAG-RUNX1 or FLAG-RUNX2 in response to dox.
**A-B**. Western blot analysis demonstrating effective silencing of RUNX1 by constitutive expression of shRx1$_{3'UTR}$ versus a non-specific (*NS*) shRNA. Dox treatment for 48h induced expression of FLAG-RUNX1 and FLAG-RUNX2 in MCF7/shRx1$_{3'UTR}$/Rx1$^{dox}$ and MCF7/shRx1$_{3'UTR}$/Rx2$^{dox}$ cells, respectively. **C-E.** Cells were kept in CSS for 2 days and treated with dox and/or E2 for 48 h as indicated (C) and global changes in mRNA expression was profiled in triplicate using the HumanHT-12 v4 BeadChip (Illumina). Venn Diagram (D) and volcano plots (E) demonstrate far more robust response to RUNX2 as compared to RUNX1.
**F-G.** Scatter plots for the E2-responsive genes (fold-change >1.2-fold, FDR<0.05 in both the MCF7/shRx1$_{3'UTR}$/Rx1$^{dox}$ and MCF7/shRx1$_{3'UTR}$/Rx2$^{dox}$ cells) demonstrate strong attenuation of the global E2 response by RUNX2 (slope=0.6624, r=0.7223), but not by RUNX1 (slope=0.8865, r=0.9635).

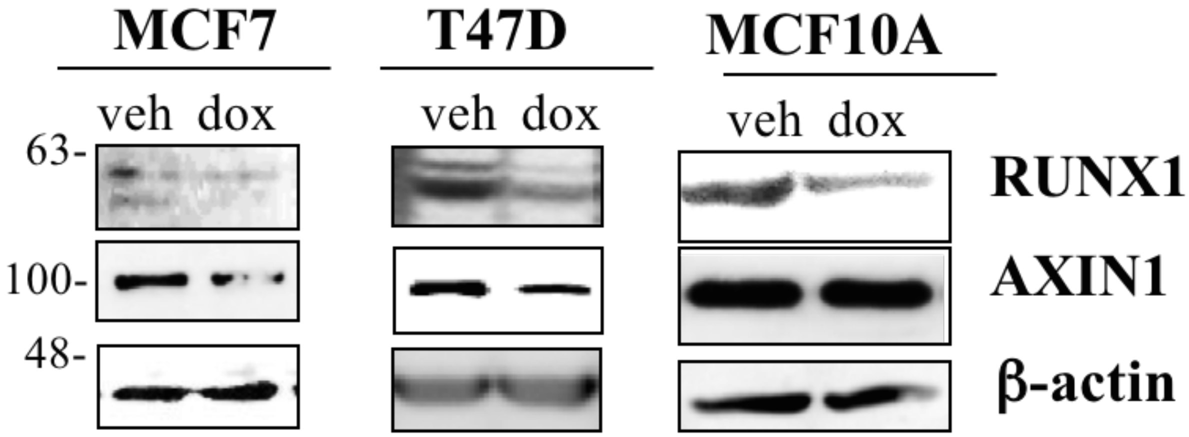


**Supplementary Fig. 4.** MCF7, T47D ($ER^+$) and MCF10A ($ER^-$) cells were engineered to express $shRx1_{RUNT}$ upon doxycycline treatment. Cells were treated with 250 ng/ml dox for 4 days and whole cell extracts were subjected to western blot analysis of the indicated proteins.

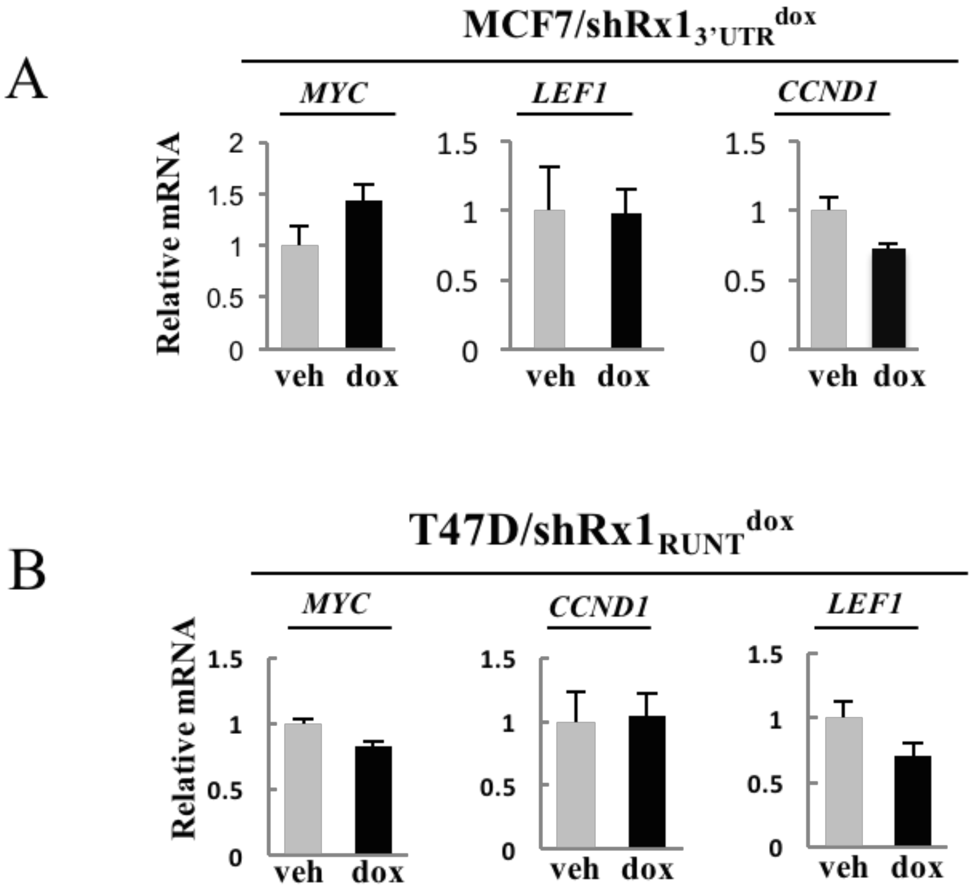


**Supplementary Fig. 5.** MCF7/shRx1$_{3'UTR}^{dox}$ (A) and T47D/shRx1$_{RUNT}^{dox}$ (B) cells were treated with 250 ng/ml dox for 48h and total RNA was subjected to RT-qPCR analysis of the indicated mRNAs. Data was corrected for 18S RNA and represent mean±SEM from triplicate experiments.

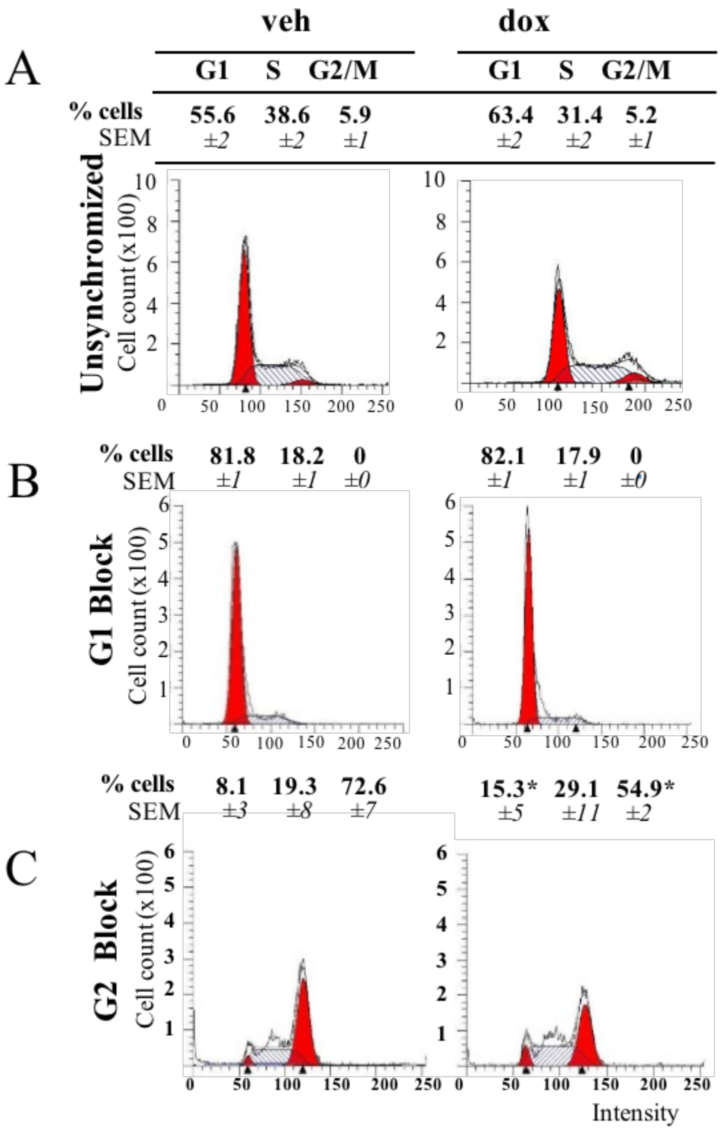


**Supplementary Fig. 6: Compromised G2/M arrest, but not G1/S arrest in MCF7/shRx1$_{RUNT}^{dox}$ cultures.**

MCF7/shRx1$_{RUNT}^{dox}$ cells were treated with dox and synchronized in order to investigate effects of RUNX1 silencing on P-b-cat as described in Figure 6H. Cells were subjected to a double thymidine block to induce a G1 block (B), which was followed by nocodazole treatment to induce a G2 block (C). The synchronized (B, C) and control cells (A) were subjected to propidium iodide staining and flow cytometry analysis to determine the percentage of cells in G1, S and G2/M. Data are Mean ± SEM from three independent experiments, with a representative cell cycle profile shown for each condition. *$p<0.05$ by t-test.

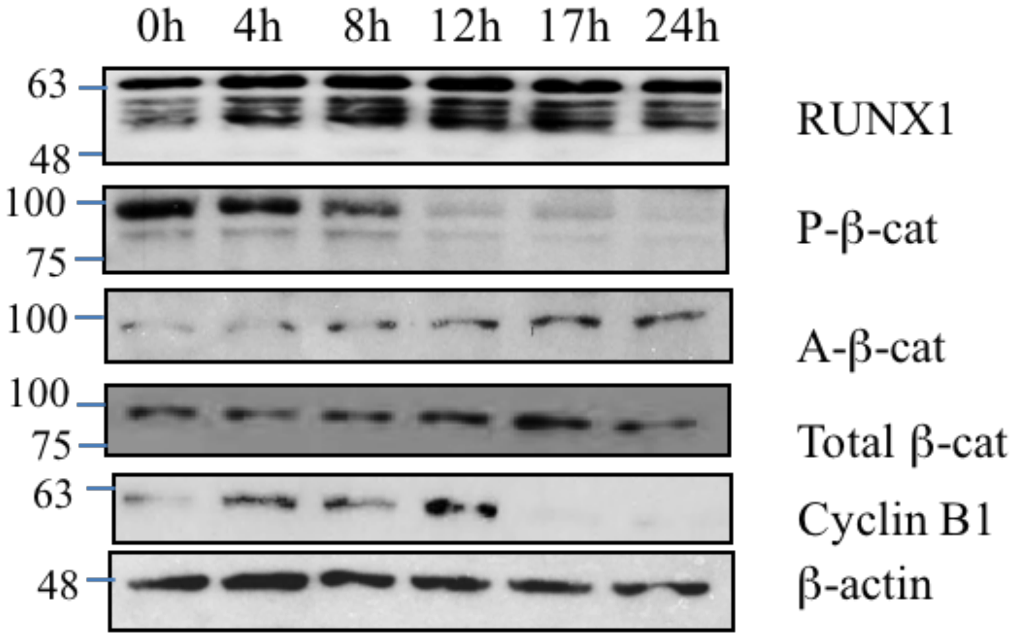


**Supplementary Fig. 7. Cell cycle-dependent P-β-catenin expression in MCF7 cells**
Western blot analysis of the indicated proteins at the indicated time points after release of MCF7 cells from a double thymidine block.

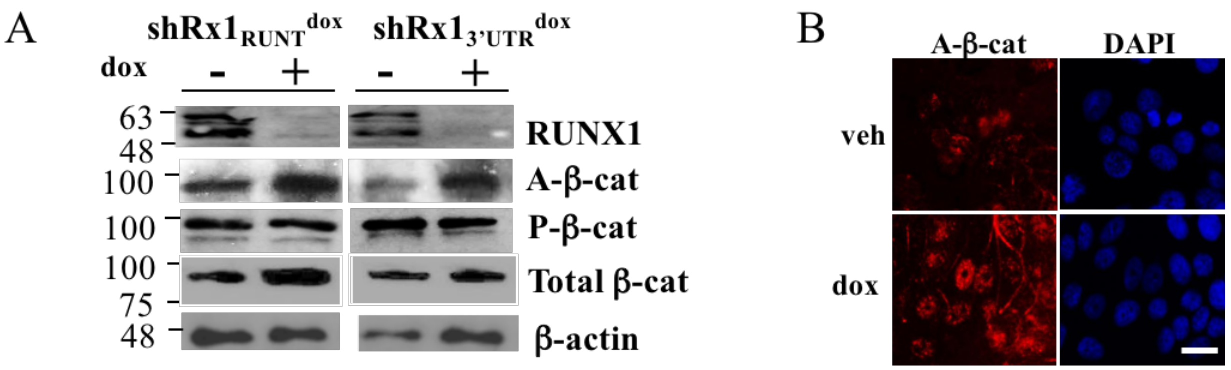


**Supplementary Fig. 8.** ***A*****,** MCF7 cells conditionally expressing either $shRx1_{RUNT}^{dox}$ (*left*) or $shRx1_{3'UTR}^{dox}$ (*right*) were treated with 250 ng/ml dox for 4 days followed by western analysis of the indicated proteins. ***B*****,** Immunofluorescence analysis of A-β-cat in MCF7/$shRx1_{RUNT}^{dox}$ cells treated with dox for 48h. Scale bar = 50 μm

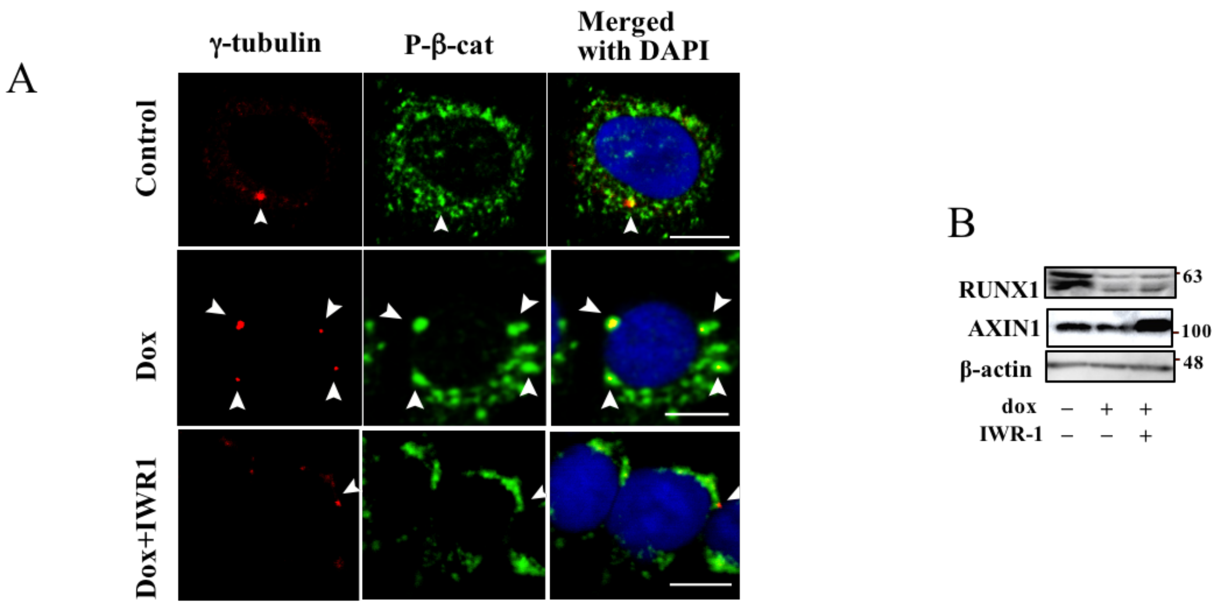


**Supplementary Fig. 9**. **Immunofluorescence imaging of P-β-cat in MCF7 cells**
MCF7/shRx1$_{RUNT}^{dox}$ cells were treated with dox for 48 hours to silence *RUNX1* and the medium was supplemented with 5μM IWR1 for the last 36h. ***A***, Centrosomal P-β-cat was identified based on co-localization with **γ**-tubulin. Immunofluorescence microscopy was carried out essentially as described previously (Liang et al. 2008, PMID:18552835). Cells grown on coverslips were fixed in methanol at -20C° and permeabilized with 0.5% Triton X-100 in PBS for 5 min. Primary antibody for P-β-cat (Cell Signaling Technology #9561) was used at a 1:200 dilution and anti-**γ**-tubulin antibody (C7604, Sigma-Aldrich) was used at a 1:200 dilution. Confocal images were acquired using a Nikon Eclipse C1 laser-scanning microscope (Nikon, PA) fitted with a 60×Nikon objective (PL APO, 1.4NA) and a Nikon image software. Scale bar = 10 μm. ***B***, Western blot analysis of the cell lysates from parallel cultures.

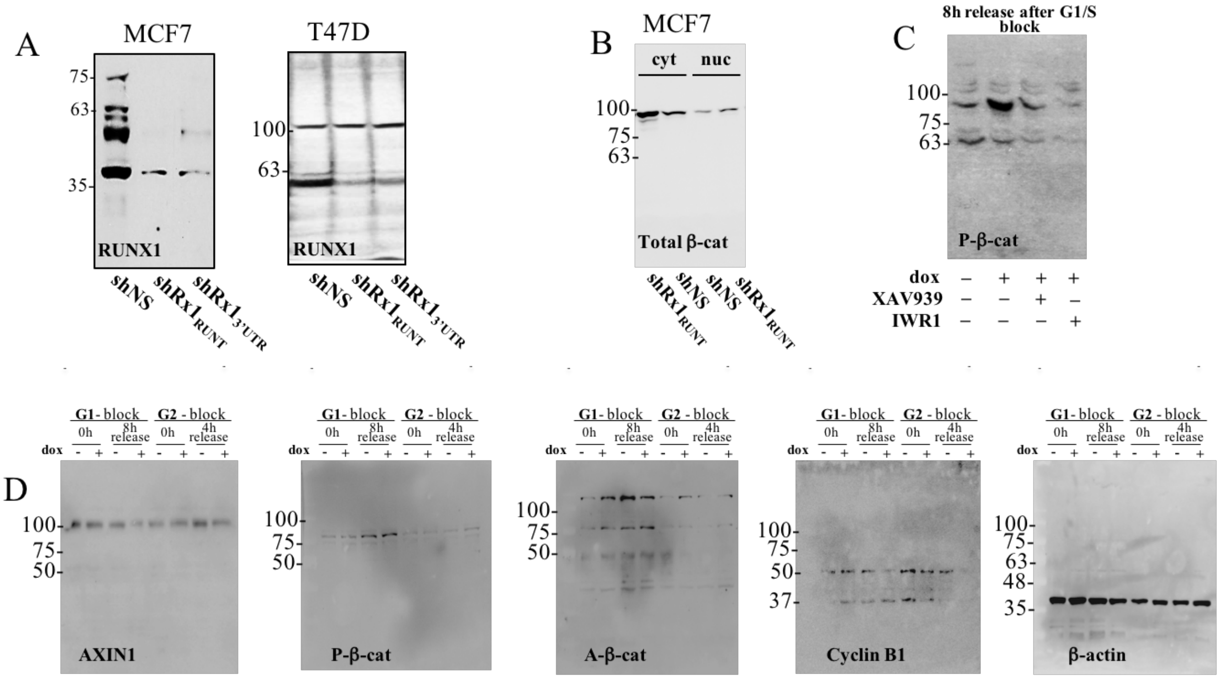


**Supplementary Fig. 10. Uncropped scans of key western blots.** (A) RUNX1 knockdown in MCF7 and T47D cells expressing a non-specific short hairpins RNA (shNS) or shRNAs targeting the RUNT domain of RUNX1 (shRx1RUNT) or its 3'UTR (shRx13'UTR). (B). Increased total b-catenin levels in nuclear and cytoplasmic protein extracts from RUNX1-depleted versus control MCF7 cells. (C) AXIN stabilizers reverse effect of RUNX1 depletion on P-β-cat in late S-phase. (D). Effect of RUNX1 knockdown on expression of the indicated proteins in the indicated phases of the cell cycle.

**Supplementary Table 1. Associations between RUNX1 and AXIN1 status, controlling for ER intensity (p-values based on the Pearson chi-square test, for the 2x2 table)**

| ER low | | | ER high | | |
|---|---|---|---|---|---|
| RUNX1 Status | AXIN1 Status | | RUNX1 Status | AXIN1 Status | |
| | Negative | Positive | | Negative | Positive |
| Negative | 2 | 7 | Negative | 2 | 1 |
| Positive | 7 | 6 | Positive | 0 | 6 |
| Odds Ratio = 0.24<br>95% CI: (0.036, 1.66)<br>p=0.15 | | | Odds Ratio = 21.7<br>95% CI: (0.64, 730.0)<br>p=0.033 | | |
| p-value for comparing the 2 odds ratio's (0.24 and 21.7) is p=0.006 – suggesting that the association between RUNX1 and AXIN1 is different, depending on the intensity of ER expression | | | | | |

**Supplementary Table 2 Sequences of oligonucleotides used in this study**

**For dox-inducible pSLIK-RUNX1 vector**

| | |
|---|---|
| F-Flag-mRx1 | GACTACAAAGACGATGACGACAAGCGTATCCCCGTAGATGCCAG |
| R-mRx1_MfeI | CTCAGCCAATTGTCAGTAGGGCCGCCACACGGCCTCC |

**For dox-inducible pSLIK shRUNX1 targeting the RUNT domain**

| | |
|---|---|
| T_h_Rx1_Runt | AGCGACCTCGAAGACATCGGCAGAAATAGTGAAGCCACAGATGTATTTCTGCCGAT GTCTTCGAGG |
| B_h_Rx1_Runt | ggcaCCTCGAAGACATCGGCAGAAATACATCTGTGGCTTCACTATTTCTGCCGATGTC TTCGAGGT |

**For dox-inducible pSLIK shRUNX1 targeting the 3'UTR region**

| | |
|---|---|
| T_h_Rx1_3UTR | AGCGCCGCTTGGTTATCCAGAAGTATTAGTGAAGCCACAGATGTAATACTTCTGGA TAACCAAGCGA |
| B_h_Rx1_3UTR | ggcaTCGCTTGGTTATCCAGAAGTATTACATCTGTGGCTTCACTAATACTTCTGGATA ACCAAGCGG |

**Primer sequences for RT-qPCR**

| Gene | Forward primer | Reverse primer |
|---|---|---|
| *GAPDH* | F: 5' -AGCCACATCGCTCAGACAC- 3' | R: 5' -GCCCAATACGACCAAATCC- 3' |
| *18S* | F: 5' -GTAACCCGTTGAACCCCATT-3' | R: 5' -CCATCCAATCGGTAGTAGCG- 3' |
| *AXIN1* | F: 5' -CAAGCAGAGGTATGTGCAGGA- 3' | R: 5' -CACAACGATGCTGTCACACG- 3' |
| *AXIN2* | F: 5' -AAACGCAATGGGAAAGGCAC- 3' | R: 5' -TGTGCTTTGGGCACTATGGG- 3' |
| *CCND1* | F: 5' -CTCCTGTGCTGCGAAGTGG- 3' | R: 5' -CTTCTGTTCCTCGCAGACCTCC- 3' |
| *LEF1* | F: 5' -ATCACACCCGTCACACATCC | R: 5' -TGGGAAAACCTGGACATGGA |
| *c-Myc* | F: 5' -AGAGAAGCTGGCCTCCTACC- 3' | R: 5' -CGTCGAGGAGAGCAGAGAAT- 3' |
| *SOX2* | F: 5' -TCAGGAGTTGTCAAGGCAGAG- 3' | R: 5' -AGAGGCAAACTGGAATCAGGA- 3' |
| *NANOG* | F: 5' -ACCTCAGCTACAAACAGGTGA- 3' | R: 5' -CTTCTGCGTCACACCATTGC- 3' |
| *ALDH1A3* | F: 5' -TGGCACGAATCCAAGAGTGG- 3' | R: 5' -TTGTCCACGTCGGGCTTATC- 3' |
| *CD44* | F: 5' -TCCCTGGATCACCGACAGCACA- 3' | R: 5' -CCTCTTGGTTGCTGTCTCAGTTGCT- 3' |
| *Axin1 (mouse)* | F: 5' -TGTCCAGTGATGCTGACACG-3' | R: 5' -AAGTGCGAGGAATGTGAGGTA- 3' |

**Primer sequences for ChIP-qPCR**

| Gene region | Forward primer | Reverse primer |
|---|---|---|
| *AXIN1* | F: 5' -CAGGTGATTTCTCGGTCACTC- 3' | R: 5' -AGGCCTCCTAGGCACAG- 3' |
| *Negative* | F: 5' -ATGGTTGCCACTGGGGATCT- 3' | R: 5' -TGCCAAAGCCTAGGGGAAGA- 3' |
